# Supplementary material for: Association of Healthy Predominantly Plant-Based Diet with Reduced Cardiovascular Disease Incidence and Mortality and Development of Novel Heart-Protective Diet Index
Source: Nutrients. 2025 Aug 19;17(16):2675. doi: 10.3390/nu17162675 (PMC12389456; doi:10.3390/nu17162675)
Supplement: Supplementary file 1 [file nutrients-17-02675-s001.zip › nutrients-3795001-supplementary.pdf]

Association of a predominantly healthy plant-based heart-protective diet with reduced cardiovascular disease incidence and mortality: findings from the UK Biobank Study

Tian Wang, Andrea Nova, Sophie Cassidy, Katherine M Livingstone, Teresa Fazia, Sayan Mitra, Cynthia M Kroeger, Andrius Masedunskas, Luisa Bernardinelli, Walter C Willett, Luigi Fontana

Table of Contents

Supplementary methods 3

Description of the UK Biobank....3

Individuals excluded from our study....3

The online 24-hour dietary measurement (the Oxford WebQ)....3

Summary of current main five plant-based diet quality indices using data from 24-hour recall....3

Calculation of Heart-Protective Diet Score....4

Validity and reliability of Heart-Protective Diet Score....4

Covariates....5

Table S1. Number of participants with available dietary data for each food item to calculate heart-protective diet score considering baseline and following instances. 7

Table S2. Characteristics of participants included (n=192,274) and excluded (n=310,120) in this study. 9

Table S3. Association between quartiles of heart-protective diet score and risk of incident cardiovascular diseases considering baseline and first two recalls. 11

Table S4. Association between quartiles of heart-protective diet score and risk of cardiovascular mortality using baseline and first two recalls. 13

Table S5. Association between quartiles of heart-protective diet score and risk of cardiometabolic abnormalities using baseline and first two recalls. 15

Table S6. A summary of the main five plant-based diet quality indices 16

Table S7. Ascertainment of CVD outcomes (ICD and UK Biobank field codes)....18

Table S8. Association between quartiles of heart-protective diet score and risk of incident cardiovascular diseases. 19

Table S9. Association between quartiles of heart-protective diet score and risk of cardiovascular mortality. 21

Table S10. Association between quartiles of heart-protective diet score and risk of cardiometabolic abnormalities. 23

Table S11. Stratified analyses for statistically significant interactions between heart-protective diet score and sex or Townsend deprivation index. 24

Table S12. Sensitivity analyses: association between quartiles of heart-protective diet score and risk of incident cardiovascular diseases and cardiometabolic abnormalities excluding self-reported diagnoses. 32

Table S13. Association between categorized food groups and risk of cardiovascular disease incidence and mortality. 34

Table S14. Food groups and nutrient intake of LIVEPLUS participants at baseline based on quartile categories of heart-protective diet scores 38

Table S15. Food groups and nutrient intake LIVEPLUS participants at one month based on quartile categories of heart-protective diet scores........39

Figure S1. Flow diagram of participants included in the study........40

Figure S2. Distribution of the heart-protective diet score........41

Figure S3. Stratified analyses for statistically significant interactions between heart-protective diet score and sex. 45

Figure S4. Stratified analyses for statistically significant interactions between heart-protective diet score and Townsend deprivation index........49

Figure S5. Sensitivity analysis: association between quartiles of heart-protective diet score and risk of incident cardiovascular diseases and cardiometabolic abnormalities including and excluding self-reported diagnoses........51

Supplementary methods

Description of the UK Biobank

The UK Biobank is an extensive prospective cohort study conducted within the general population, which enrolled over 500,000 individuals aged 40-69 years during the period from 2006 to 2010 [1]. As part of their participation, the UK Biobank participants were required to complete a touch-screen questionnaire that gathered information about their sociodemographic characteristics and lifestyle factors. Additionally, they provided biological samples, including blood samples, and underwent various anthropometric measurements as part of the study protocol [1]. The UK Biobank was approved by the North-West Multi-Centre Research Ethical Committee (REF: 11/NW/03820). This study complies with the Declaration of Helsinki. All individuals had provided written informed consent to participate in the study.

Individuals excluded from our study

We excluded individuals who had: (i) missing or implausible data regarding total energy intake (men: <800 or >4200 kcal/day; n=2030; women: <600 or >3500 kcal/day; n=2177), and (ii) prevalent cases of CVD at the time of recruitment (n=13,008).

The online 24-hour dietary measurement (the Oxford WebQ)

Dietary information was collected during the recruitment phase using a self-completed touchscreen questionnaire that assessed the frequency of consumption of main food items. A significant subset of participants (n=211,050) completed at least one web-based 24-hour dietary measurement using the Oxford WebQ tool between 2009 and 2012. The Oxford WebQ is an online 24-hour dietary measurement tool, which collected consumption data of 206 types of foods and 32 types of drinks during the previous 24 hours. Standard categories and descriptions (e.g., slices of bread, slices of bacon) were used to assist participants in portion sizes estimation of each item. Example questions of the Oxford WebQ include: "Did you have any of these yesterday?", "How much of the following did you drink yesterday?", or "Would you say that what you ate and drank yesterday was fairly typical for you? (yes/no)". The results generated from the Oxford WebQ has captured similar food items and nutrient intake estimates in comparison to an interviewer-administrated dietary assessment, with moderate-to-strong correlations for most nutrients (Spearman's correlation coefficients between 0.5 and 0.9)[2]. This indicates a reasonably good agreement between the two methods in capturing dietary information and nutrient intake.

A previous study by Bradbury *et al.* evaluated the repeatability of the touchscreen questionnaire in the UK Biobank cohort and found a generally good agreement between reported consumption at baseline and approximately 4 years later[3]. These included fish and meat items (>90% reported the same or adjacent category of intake) and vegetable and fruit (>72% reported the same or adjacent category of intake), with weighted K coefficient showing substantial agreement for oily fish, beef and lamb, poultry, processed meat and fresh fruit. This study also showed good agreement for all foods and food groups intake in participants who completed 24-h recall for more than once. In the present study, we calculated the "heart-protective diet score at baseline" and the "averaged heart-protective diet score" based on repeated measurements and examined the association between them and found a moderate correlation (Pearson correlation coefficient=0.53; p-value < 0.001) between these two scores. Therefore, for participants who completed the dietary measurement multiple times, we used the earliest data to maximize the follow-up duration.

Summary of current main five plant-based diet quality indices using data from 24-hour recall

Marchese et al.[15] recently reviewed 35 plant-based diet quality indices, of which 19, 9 and 5 indices were developed based on FFQ, semi-FFQ and 24hr recall, respectively. Both FFQ and 24hr recall are commonly used dietary assessment approaches in observational studies to reflect participants' dietary patterns. However, as

highlighted by Nutritools and Dietary Assessment Guidelines[16], short FFQs are not reliable for evaluating total diet, and FFQ in general is prone to inaccurate or subjective reporting. Thus, to allow a thorough evaluation of diet quality and answer the mixed findings by previous literature, detailed dietary data is needed to reflect individuals' adherence to a plant-based diet. Table S5 summarize the current main five plant-based diet quality indices utilizing data from 24hr recall. Previously validated plant-based diet index[9, 23] (hPDI and uPDI) only evaluates the quality of plant-based food items but assigns all animal food items negative scores. Only three indices (Comprehensive Diet Quality Index (cDQI), Diet Quality Index Associated to the Digital Food Guide (DQI-DFG) [34], and Global Diet Quality Score (GDQS) [35]) consider healthiness of both animal-based and plant-based foods. Discrepancies in categorization of food groups among these scores are identified in red meats, eggs, processed meats, full-fat dairy products, tubers and roots, refined carbohydrates and sugars and sweets. For example, red meats, refined grains and sweets are included as 'moderation components' groups by DQI-DFG, and all dairy products including full-fat dairy and cream are categorized as 'adequacy components', while these food groups are discouraged by international guidelines[20-22] with an encouragement of the use of reduced-fat dairy products; cDQI considers 'eggs' as unhealthful animal groups while the other two scores put 'eggs' in healthy food groups, while international guidelines did not encourage or discourage the consumption of eggs. None of these scores fully reflect the diet recommended by AHA, HF and ESC/EAS guidelines[20-22] for the prevention and management of cardiovascular disease.

Calculation of Heart-Protective Diet Score

To derive the HPDS, we categorized the food and drink items into 22 food groups (Table 1) and ranked consumption into quintiles. The amount of food and drink items consumed was converted in grams or ml based on the Eatwell Guide by the UK National Health Service [27]. Table S7 displays the number of participants with data available for each food item. For food groups associated with positive scores, a score of 5 was assigned to individuals in the highest quintile of consumption, while a score of 1 was given to those in the lowest quintile. Conversely, for food groups linked to negative scores, a score of -1 was assigned to participants in the lowest quintile, and a score of -5 was given to those in the highest quintile. A score of 0 was assigned when a participant reported not consuming a particular food item. The sum of scores for the 22 groups generated the HPDS, reflecting diet quality in terms of cardiovascular health.

Validity and reliability of Heart-Protective Diet Score

Our HPDS were evaluated by these approaches: i). construct validity[15, 28]: the association between HPDS and foods and nutrient intakes, and the difference of HPDS by sociodemographic characteristic; ii). criterion validity[28]: the association of HPDS and clinical outcomes; iii). reliability[15, 28] was evaluated by Cronbach's Alpha (0.5 or greater is considered to have good internal consistency) method[29] in 5419 participants with all five instances of 24-hour recalls, with a result of 0.79 [95%CI: 0.78, 0.81] showing good internal consistency.; and iv). content validity: we employed the expert panel consensus approach to validate the HPDS, where the draft HPDS were circulated and reviewed by eight subject experts (two professors, six PhD degree holders, two practitioners, two dietitians and three exercise physiologists) of different related disciplines (Nutrition and Food Science, Epidemiology, Cardiology, Life Science, Psychology, and Statistics). In addition, the HPDS was validated in a clinical trial delivered in individuals with cardiovascular disease at two different time points (shown in Table S14 and Table S15). We calculated the HPDS at baseline, and for participants with repeated measurements we calculated the averaged HPDS scores to account for variations over time. For participants with missing baseline data but with subsequent assessments, the earliest assessment served as baseline to maximize sample size and follow-up duration.

Covariates

To adjust for confounding, we included covariates in the models for each outcome: age at recruitment (determined from dates of birth and baseline assessment), sex, ethnicity (white, non-white), education (college or university degree, yes), average total household income before tax (< £18000, £18000-30999, £31000- £51999, £52000-100000, or > £100000), Townsend Social Deprivation Index, lifestyle factors (including physical activity, sitting, sleep quality and smoking habits), dietary supplement use (including vitamin, mineral and other dietary supplements), history of hyperglycaemia or T2DM diagnosis (ICD10: R739, E11) and history of cancer diagnosis (excluding malignant neoplasm of skin) (ICD10: CXX [excluding C449], D0X) at baseline. The Townsend Social Deprivation Index[4], created by the UK Biobank group, generates a composite measure of deprivation based on four variables: unemployment, non-car ownership, non-home ownership, and household overcrowding. A negative value of the Townsend Social Deprivation Index corresponds to high socio-economic status. For physical activity, sitting and sleep quality, we created a score as follows:

1. *Physical activity score*

Participants' self-reported answers on International Physical Activity Questionnaire (IPAQ)[5] based questions were used to calculate physical activity score as “Low”, “Moderate”, or “High”. The number of days in a week with walking 10+ mins, moderate activity 10+ mins and vigorous activity 10+ mins, and the duration of that activity in a typical day, were used to derive the metabolic equivalent of task (MET) minutes/week for each activity[6], as well as for outliers for which the total sum duration of walking, moderate and vigorous physical activity was > 960 mins/day. Responses of < 10 mins and their associated days were re-coded to zero (minimum values for duration activities). Walking, moderate, and vigorous physical activity time, when >180 mins, were truncated to be equal to 180 mins. Following IPAQ algorithm: a “High” score was assigned if the vigorous-intensity activity was performed on ≥3 days with total MET-minutes/week ≥1500 OR a combination of walking, moderate- or vigorous- intensity activities was performed 7 days accumulating ≥3000 MET-minutes/week; a “Moderate” score was assigned if vigorous-intensity activity was performed on ≥3 days and ≥ 20mins/day OR a moderate intensity activity was performed ≥5 days and/or walking of ≥30 mins/day OR a combination of walking, moderate- or vigorous- intensity activities was performed ≥5 days achieving ≥600 MET-minutes/week; a “Low” score was assigned in all the other cases (i.e., none or insufficient activity). Physical scores for subjects who answered “Do not know” or “Prefer not to answer” for any question used to build the score were set as missing values.

2. *Sitting score*

Sitting score was calculated based on the total sum of reported answers about time (hours/day) of i) watching TV, ii) using the computer, iii) driving. Sitting scores for subjects who answered “Do not know” or “Prefer not to answer” for any question used to build the score were set as missing values. Finally, we categorized the score in tertiles.

3. *Sleep score*

Sleep score was calculated based on reported answers about sleep duration (hours/day), chronotype (e.g., “morning” or “evening” person), sleeplessness/insomnia (i.e., “never/rarely”, “sometimes”, “usually”), snoring (“yes”, “no”), and daytime dozing/sleeping (narcolepsy) (i.e., “never/ rarely”, “sometimes”, “often”, “all of the time”). This approach was already used in the UKBB[7]. Reporting an early chronotype (i.e., “morning” or “morning than evening”), ≥7 hours/day of sleep, never or rarely insomnia symptoms, no snoring and no (i.e., never/rarely or sometimes) daytime sleepiness correspond to have low-risk sleep factors. For each factor, participants were scored as 1 if they had it and 0 if they did not have it. All factors were then summed to obtain the overall sleep score. Sleep

scores for subjects who answered “Do not know” or “Prefer not to answer” for any question used to build the score were set as missing values.

Table S1. Number of participants with available dietary data for each food item to calculate heart-protective diet score considering baseline and following instances.

Total	Baseline	First instance	Second instance	Third instance	Fourth instance
192274	63408 (33%)	72707 (38%)	25019 (13%)	18316 (9%)	12824 (7%)

Food groups	Baseline N (%)	At least one instance including baseline data N (%)
Wholegrains	69426 (13.8%)	209215 (41.6%)
Fruits	70684 (14.1%)	210954 (42.0%)
Non-starchy Vegetables	70684 (14.1%)	210954 (42.0%)
Starchy Vegetables	70684 (14.1%)	210954 (42.0%)
Nuts & Seeds	70684 (14.1%)	210954 (42.0%)
Legumes & Beans, Other Vegetarian Protein Alternatives	70684 (14.1%)	210954 (42.0%)
Uncoated Fish & Seafood	70684 (14.1%)	210954 (42.0%)
Eggs	70684 (14.1%)	210954 (42.0%)
(Reduced-fat and/or No Added Sugar) Milk & Dairy Products	70684 (14.1%)	210954 (42.0%)
Tea, Coffee & Other Low-calorie Drinks	70684 (14.1%)	210954 (42.0%)
Homemade soup	70684 (14.1%)	210954 (42.0%)
Refined grains & cereals, including discretionary choices	69426 (13.8%)	209215 (41.6%)
Potatoes	70684 (14.1%)	210954 (42.0%)
Meat, Poultry & Processed Meat	70684 (14.1%)	210954 (42.0%)
Coated Fish & Seafood	70684 (14.1%)	210954 (42.0%)
(Full-fat and/or Added Sugar) Milk & Dairy Products	70684 (14.1%)	210954 (42.0%)
Processed Soup	70684 (14.1%)	210954 (42.0%)
Sugar, Sweets & Desserts, Cookies & Pastries	70684 (14.1%)	210954 (42.0%)
Savoury Snacks	70684 (14.1%)	210954 (42.0%)

Sugary Drinks including Juices & Sugar-sweetened Beverages	70684 (14.1%)	210954 (42.0%)
Artificial sweeteners	70684 (14.1%)	210954 (42.0%)
Unhealthy Fat (with/without Carbohydrates)	70684 (14.1%)	210954 (42.0%)

Table S2. Baseline characteristics of participants included (n=192,274) and excluded (n=310,120) in this study.

	Included (n=192274)		Excluded (n=310120)	
	**Total**		**Total**	
**Characteristics**	**N**	**Data**	**N**	**Data**
Age, years	192274	56.3 (7.9)	310117	57.5 (8.2)
Sex, male	192274	43.3%	310120	47.0%
Ethnicity, White	189733	95.6%	303866	93.2%
Townsend Deprivation Index	192028	-1.6 (2.9)	309740	-1.1 (3.2)
College or university degree, Y	191371	43.4%	300891	25.1%
Average total household income before tax, < £18000	172512	13.2%	252740	23.2%
Smoking habit, current	191802	7.7%	307642	12.3%
Physical activity score, never	158917	10.4%	227076	10.3%
Sitting score (hours/day)	189448	4.7 (2.3)	293906	4.9 (2.5)
Sleep score	160974	3.7 (1.0)	249634	3.6 (1.0)
Heart-protective diet score	192274	2.9 (7.7)	16941	1.8 (8.2)
BMI, kg/m2	191785	26.8 (4.6)	307502	27.8 (4.9)
SBP, mmHg	185097	136.6 (18.3)	287180	138.6 (18.9)
DBP, mmHg	185100	81.9 (10.0)	287182	82.4 (10.3)
TC, mmol/L	182092	5.8 (1.1)	288648	5.6 (1.2)
LDL-C, mmol/L	181789	3.6 (0.8)	288113	3.53 (0.9)
HDL-C, mmol/L	167016	1.5 (0.4)	265025	1.4 (0.4)
Triglycerides, mmol/L	181967	1.7 (1.0)	288403	1.79 (1.1)
HbA1c (mmol/mol)	180738	35.4 (5.8)	287067	36.6 (7.3)

Data are expressed as mean (SD) or %. HPDS, Heart-protective diet score; BMI, Body Mass Index; SBP, Systolic Blood Pressure; DBP, Diastolic Blood Pressure; HDL-C, high-density lipoprotein cholesterol; LDL-C, low-density lipoprotein cholesterol; TC, total cholesterol; HbA1c, glycated haemoglobin

Table S3. Association between quartiles of heart-protective diet score and risk of incident cardiovascular diseases considering baseline and first two recalls.

Outcome	HR [95% CI]	Unadjusted p-value
**CVD Incidence**		
Q1 (Ref.)	1.00	
Q2	0.98 [0.94,1.03]	0.417
Q3	0.95 [0.90,0.99]	0.023
Q4	0.92 [0.87,0.96]	<0.001
**IHD Incidence**		
Q1 (Ref.)	1.00	
Q2	0.96 [0.91,1.01]	0.134
Q3	0.92 [0.87,0.98]	0.006
Q4	0.88 [0.83,0.93]	<0.001
**MI Incidence**		
Q1 (Ref.)	1.00	
Q2	0.96 [0.87,1.06]	0.424
Q3	0.91 [0.82,1.01]	0.073
Q4	0.80 [0.72,0.89]	<0.001
**Stroke Incidence**		
Q1 (Ref.)	1.00	
Q2	0.95 [0.88,1.07]	0.323
Q3	0.91 [0.81,0.99]	0.098
Q4	0.97 [0.87,1.07]	0.567
**HF Incidence**		
Q1 (Ref.)	1.00	
Q2	0.95 [0.86,1.05]	0.317
Q3	0.90 [0.80,1.00]	0.044
Q4	0.83 [0.74,0.93]	0.001
**AF Incidence**		
Q1 (Ref.)	1.00	

Q2	1.04 [0.97,1.11]	0.242
Q3	1.00 [0.94,1.07]	0.957
Q4	0.98 [0.92,1.05]	0.617

Cox proportional hazard models were adjusted by age (timescale), sex, ethnicity, Townsend deprivation index, average house income, education level, dietary supplement use, history of cancer diagnosis, history of hyperglycemia/type 2 diabetes diagnosis, physical activity level, sitting, sleep quality, smoke habits. AF, Atrial Fibrillation; CVD, Cardiovascular Disease; HF, Heart Failure; HR, Hazard Ratio; IHD, Ischemic Heart Disease; MI, Myocardial Infarction.

Table S4. Association between quartiles of heart-protective diet score and risk of cardiovascular mortality using baseline and first two recalls.

Outcome	HR [95% CI]	Unadjusted p-value
**CVD Mortality**		
Q1 (Ref.)	1.00	
Q2	0.82 [0.69,0.97]	0.023
Q3	0.87 [0.73,1.03]	0.107
Q4	0.74 [0.61,0.89]	0.001
**IHD Mortality**		
Q1 (Ref.)	1.00	
Q2	0.86 [0.67,1.11]	0.242
Q3	0.93 [0.72,1.20]	0.559
Q4	0.73 [0.55,0.97]	0.029
**MI Mortality**		
Q1 (Ref.)	1.00	
Q2	0.71 [0.51,1.01]	0.054
Q3	0.81 [0.58,1.14]	0.225
Q4	0.65 [0.45,0.95]	0.025
**Stroke Mortality**		
Q1 (Ref.)	1.00	
Q2	0.83 [0.60,1.15]	0.254
Q3	0.91 [0.66,1.26]	0.573
Q4	0.84 [0.60,1.17]	0.304
**HF Mortality**		
Q1 (Ref.)	1.00	
Q2	0.66 [0.41,1.05]	0.078
Q3	0.69 [0.43,1.12]	0.132
Q4	0.52 [0.31,0.90]	0.018
**AF Mortality**		
Q1 (Ref.)	1.00	
Q2	NE	NE

Q3	NE	NE
Q4	NE	NE

Cox proportional hazard models were adjusted by age (timescale), sex, ethnicity, Townsend deprivation index, average house income, education level, dietary supplement use, history of cancer diagnosis, history of hyperglycemia/type 2 diabetes diagnosis, physical activity level, sitting, sleep quality, smoke habits. AF, Atrial Fibrillation; CVD, Cardiovascular Disease; HF, Heart Failure; HR, Hazard Ratio; IHD, Ischemic Heart Disease; MI, Myocardial Infarction; NE, Not Estimable.

*Adjusted p-values were obtained using Benjamini-Hochberg procedure.

Table S5. Association between quartiles of heart-protective diet score and risk of cardiometabolic abnormalities using baseline and first two recalls.

Outcome	HR [95% CI]	Unadjusted p-value
**Overweight/Obesity**		
Q1 (Ref.)	1.00	
Q2	0.89 [0.84,0.94]	<0.001
Q3	0.85 [0.80,0.90]	<0.001
Q4	0.79 [0.74,0.84]	<0.001
**Hyperlipidaemia**		
Q1 (Ref.)	1.00	
Q2	1.01 [0.90,1.13]	0.826
Q3	0.92 [0.81,1.03]	0.142
Q4	0.85 [0.75,0.97]	0.012
**Hypertension**		
Q1 (Ref.)	1.00	
Q2	0.98 [0.94,1.03]	0.418
Q3	0.91 [0.87,0.95]	<0.001
Q4	0.92 [0.88,0.96]	<0.001
**Hyperglycaemia/Type 2 Diabetes**		
Q1 (Ref.)	1.00	
Q2	0.87 [0.81,0.93]	<0.001
Q3	0.83 [0.78,0.89]	<0.001
Q4	0.84 [0.78,0.90]	<0.001

Cox proportional hazard models were adjusted by age (timescale), sex, ethnicity, Townsend deprivation index, average house income, education level, dietary supplement use, history of cancer diagnosis, history of hyperglycemia/type 2 diabetes diagnosis (except when hyperglycemia/type 2 diabetes was the outcome), physical activity level, sitting, sleep quality, smoke habits. HR, Hazard Ratio.

Table S6. A summary of the main five plant-based diet quality indices

Index name	Country	Food groups	Calculation
Animal-based diet quality index (aDQI) [8]	U.S.	6 food groups<br><br>Healthy animal groups: fish/seafood, poultry and dairy products<br><br>Unhealthful animal groups: red meats, eggs and processed meats	Dietary intake of each food component is adjusted for total energy intake (EI) using density method, divided into sex-specific quintile cut offs, and each quintile scored from 0 to 5. Intake of each food group adjusted for total EI using density method. EI adjusted in analysis models.
Comprehensive Diet Quality Index (cDQI) [8]	U.S.	17 food groups<br><br>Healthy plant groups: wholegrains, vegetables (excluding white potatoes), whole fruits, nuts/seeds/legumes, vegetable oils, and coffee/tea. Unhealthful plant group: refined grains, fruit juices, white potatoes, sugar-sweetened beverages (SSBs), and sweets/desserts<br><br>Healthy animal groups: fish/seafood, poultry and dairy products. Unhealthful animal groups: red meats, eggs and processed meats	Same as aDQI. Healthy animal and plant foods positively scored; unhealthy animal and plant foods reverse scored. EI adjusted in analysis models.
Diet Quality Index Associated to the Digital Food Guide (DQI-DFG) [9]	Brazil	11 food groups<br><br>Moderation components: sugars and sweets; meats: beef, pork and processed meat; refined cereals, and processed fats<br><br>Adequacy components: poultry, fish and eggs, whole cereals, tubers and roots, fruits, vegetables, legumes and oilseeds, milk and dairy products, and oils and fats	All food groups scored a maximum of 5 or 10, on the basis of portion per 1,000 kcal. Dichotomous scoring for moderation components, where the maximum score is awarded for being within the recommended intake range or scored 0 when this range exceeded. For adequacy components, increasing score for higher intake with the maximum score awarded for being within or above the recommended intake range. For whole cereals, tubers and roots, fruits, and milk and dairy products with intake above the

			recommendation, decreasing scores were given for intake that is twice the upper limit, and no points for intake above twice the upper limit
Global Diet Quality Score (GDQS) [10]	African countries, China, India, Mexico, US	25 food groups<br><br>Healthy food groups: citrus fruits, deep orange fruits, other fruits, dark green leafy vegetables, cruciferous vegetables, deep orange vegetables, other vegetables, deep orange tubers, legumes, nuts and seeds, whole grains, liquid oils, fish and shellfish, poultry and game meat, low-fat dairy, and eggs. Unhealthy groups when consumed in excess: high-fat dairy and red meat.<br><br>Unhealthy food groups: processed meat, refined grains and baked goods, sweets and ice cream, SSBs, juice, white roots and tubers, and purchased deep fried foods	All foods ranked into 3 categories of consumed amounts (g/d) (apart from high-fat dairy which has 4 categories). The categories of consumption are specific for each food group and points are assigned on the basis of these categories. Healthy foods have higher scores for higher consumption. Unhealthy when consumed in excess foods are scored in increasing points until specific amounts are consumed. Unhealthy foods are given more points for lower consumption. All groups scored from 0 to 2 points, apart from: cruciferous vegetables, deep orange vegetables, other vegetables, and deep orange tubers (0–0.5 points); red meat (0–1 point); dark green leafy vegetables, legumes, nuts and seeds (4 points). EI adjusted in some analysis models.
Plant-based Diet Quality Index (pDQI) [11]	U.S.	11 food groups<br><br>Healthy plant group: whole grains, vegetables (excluding white potatoes), whole fruits, nuts/seeds/legumes, vegetable oils, and coffee/tea<br><br>Unhealthful plant group: refined grains, fruit juices, white potatoes, SSBs, and sweets/desserts	Dietary intake of each food component was adjusted for total EI using density method, divided into sex-specific quintile cut offs, and each quintile scored from 0 to 5. Healthy plant foods positively scored, and unhealthy plant foods reverse scored. EI adjusted in analysis models.

Table S7. Ascertainment of CVD outcomes (ICD and UK Biobank field codes)

**Disease**	**ICD10 codes**	**UK Biobank Field for diagnosis date**	**UK Biobank Field for source of diagnosis***	**Diagnosis from Electronic Health Records**
**Cardiovascular diseases (CVDs)**				
IHD	I20, I24, I25	131296 Date I20 first reported (angina pectoris)	131297 Source of report of I20 (angina pectoris)	Angina
		131304 Date I24 first reported (other acute IHDs)	131305 Source of report of I24 (other acute IHDs)	
		131306 Date I25 first reported (chronic IHD)	131307 Source of report of I25 (chronic IHD)	
MI	I21, I22, I23	131298 Date I21 first reported (acute MI)	131299 Source of report of I21 (acute MI)	MI
		131300 Date I22 first reported (subsequent MI)	131301 Source of report of I22 (subsequent MI)	
		131302 Date I23 first reported (certain current complications following acute MI)	131303 Source of report of I23 (certain current complications following acute MI)	
Stroke	I61, I62, I63, I64	131364 Date I62 first reported (other nontraumatic intracranial haemorrhage)	131365 Source of report of I62 (other nontraumatic intracranial haemorrhage)	Stroke
		131362 Date I61 first reported (intracerebral haemorrhage)	131363 Source of report of I61 (intracerebral haemorrhage)	
		131366 Date I63 first reported (cerebral infarction)	131367 Source of report of I63 (cerebral infarction)	
		131368 Date I64 first reported (stroke, not specified as haemorrhage or infarction)	131369 Source of report of I64 (stroke, not specified as haemorrhage or infarction)	
HF	I50	131354 Date I50 first reported (HF)	131355 Source of report of I50 (HF)	-
Arrhythmia and conduction disorders including AF	I48	131350 Date I48 first reported (AF and flutter)	131351 Source of report of I48 (AF and flutter)	-
**Cardiometabolic diseases**				

Overweight and Obesity	E66	130792 Date E66 first reported (obesity)	130793 Source of report of E66 (obesity)	-
Hypertension	I10	131286 Date I10 first reported (essential (primary) hypertension)	131287 Source of report of I10 (essential (primary) hypertension)	Hypertension
Hyperglycaemia/ T2DM	R739, E11	130708 Date E11 first reported (non-insulin-dependent diabetes mellitus)	130709 Source of report of E11 (non-insulin-dependent diabetes mellitus)	Type II diabetes mellitus
		41270 Diagnoses – ICD10 (to identify ICD10 R739 diagnosis)		

* Source includes primary care, death register, hospital admission and self-report.
AF, Atrial Fibrillation; CVD, Cardiovascular Disease; HF, Heart Failure; HR, Hazard Ratio; IHD, Ischemic Heart Disease; MI, Myocardial Infarction; T2DM, Type 2 diabetes mellitus.

Table S8. Association between quartiles of heart-protective diet score and risk of incident cardiovascular diseases.

Outcome	N	Events	HR [95% CI]	p-value	Adjusted p-value
**CVD Incidence**					
1 SD increase	171582	20692	0.97 [0.95, 0.98]	<0.001	<0.001
Q1 (Ref.)	42704	5743	1.00 (Ref.)		
Q2	42539	5261	0.97 [0.94, 1.01]	0.124	0.173
Q3	43200	4979	0.93 [0.90, 0.97]	<0.001	<0.001
Q4	43139	4709	0.92 [0.88, 0.95]	<0.001	<0.001
**IHD Incidence**					
1 SD increase	181033	11241	0.96 [0.94, 0.98]	<0.001	<0.001
Q1 (Ref.)	45174	3273	1.00 (Ref.)		
Q2	44931	2869	0.96 [0.91, 1.01]	0.11	0.17
Q3	45498	2681	0.93 [0.88, 0.98]	0.005	0.012
Q4	45430	2418	0.89 [0.84, 0.94]	<0.001	<0.001
**MI Incidence**					
1 SD increase	188822	3452	0.94 [0.91, 0.97]	<0.001	<0.001
Q1 (Ref.)	47377	1070	1.00 (Ref.)		
Q2	46895	905	0.97 [0.89, 1.06]	0.492	0.503
Q3	47387	792	0.9 [0.82, 0.99]	0.026	0.045
Q4	47163	685	0.85 [0.77, 0.94]	0.001	0.003
**Stroke Incidence**					
1 SD increase	189171	3103	0.97 [0.94, 1.01]	0.126	0.145
Q1 (Ref.)	47594	853	1.00 (Ref.)		
Q2	47024	776	0.95 [0.87, 1.05]	0.350	0.384
Q3	47463	716	0.88 [0.8, 0.98]	0.017	0.033
Q4	47090	758	0.96 [0.87, 1.07]	0.481	0.503
**HF Incidence**					

1 SD increase	189085	3189	0.94 [0.91, 0.98]	0.002	0.003
Q1 (Ref.)	47515	932	1.00 (Ref.)		
Q2	46980	820	0.95 [0.86, 1.04]	0.255	0.31
Q3	47443	736	0.86 [0.78, 0.95]	0.003	0.008
Q4	47147	701	0.86 [0.77, 0.95]	0.003	0.008
**AF Incidence**					
1 SD increase	183361	8913	0.99 [0.97, 1.01]	0.272	0.291
Q1 (Ref.)	46120	2327	1.00 (Ref.)		
Q2	45508	2292	1.02 [0.96, 1.08]	0.575	0.575
Q3	46000	2179	0.97 [0.91, 1.03]	0.269	0.312
Q4	45733	2115	0.97 [0.91, 1.03]	0.343	0.384

Cox proportional hazard models were adjusted by age (timescale), sex, ethnicity, Townsend deprivation index, average house income, education level, dietary supplement use, history of cancer diagnosis, history of hyperglycemia/type 2 diabetes diagnosis, physical activity level, sitting, sleep quality, smoke habits. AF, Atrial Fibrillation; CVD, Cardiovascular Disease; HF, Heart Failure; HR, Hazard Ratio; IHD, Ischemic Heart Disease; MI, Myocardial Infarction. SD=Standard Deviation.

*Adjusted p-values were obtained using Benjamini-Hochberg procedure.

Table S9. Association between quartiles of heart-protective diet score and risk of cardiovascular mortality.

Outcome	N	Events	HR [95% CI]	p-value	Adjusted p-value
**CVD Mortality**					
1 SD increase	191143	1131	0.91 [0.86, 0.97]	0.002	0.003
Q1 (Ref.)	48082	365	1.00 (Ref.)		
Q2	47528	272	0.83 [0.71, 0.97]	0.019	0.036
Q3	47914	265	0.84 [0.71, 0.98]	0.028	0.047
Q4	47619	229	0.77 [0.65, 0.91]	0.003	0.008
**IHD Mortality**					
1 SD increase	181033	11241	0.96 [0.94, 0.97]	<0.001	<0.001
Q1 (Ref.)	45174	3273	1.00 (Ref.)		
Q2	44931	2869	0.96 [0.91, 1.01]	0.117	0.17
Q3	45498	2681	0.93 [0.88, 0.98]	0.004	0.01
Q4	45430	2418	0.88 [0.84, 0.93]	<0.001	<0.001
**MI Mortality**					
1 SD increase	191982	292	0.88 [0.78, 1.00]	0.041	0.056
Q1 (Ref.)	48342	105	1.00 (Ref.)		
Q2	47733	67	0.73 [0.54, 1]	0.049	0.079
Q3	48111	68	0.79 [0.58, 1.07]	0.127	0.173
Q4	47796	52	0.66 [0.47, 0.92]	0.016	0.033
**Stroke Mortality**					
1 SD increase	191923	351	0.98 [0.88, 1.09]	0.675	0.675
Q1 (Ref.)	48345	102	1.00 (Ref.)		
Q2	47721	79	0.79 [0.59, 1.06]	0.113	0.170
Q3	48095	84	0.82 [0.61, 1.1]	0.181	0.226
Q4	47762	86	0.85 [0.63, 1.14]	0.270	0.312
**HF Mortality**					
1 SD increase	192133	141	0.85 [0.71, 1.01]	0.060	0.075
Q1 (Ref.)	48391	56	1.00 (Ref.)		
Q2	47771	29	0.59 [0.38, 0.93]	0.023	0.041
Q3	48145	34	0.73 [0.47, 1.13]	0.162	0.208
Q4	47826	22	0.52 [0.31, 0.86]	0.011	0.025
**AF Mortality**					
1 SD increase	192274	35	NE	NE	NE
Q1 (Ref.)	48437	16	1.00		
Q2	47775	3	NE	NE	NE
Q3	48007	5	NE	NE	NE
Q4	48020	11	NE	NE	NE

Cox proportional hazard models were adjusted by age (timescale), sex, ethnicity, Townsend deprivation index, average house income, education level, dietary supplement use, history of cancer diagnosis, history of hyperglycemia/type 2 diabetes diagnosis, physical activity level, sitting, sleep

quality, smoke habits. AF, Atrial Fibrillation; CVD, Cardiovascular Disease; HF, Heart Failure; HR, Hazard Ratio; IHD, Ischemic Heart Disease; MI, Myocardial Infarction; NE, Not Estimable. SD=Standard Deviation.

*Adjusted p-values were obtained using Benjamini-Hochberg procedure.

Table S10. Association between quartiles of heart-protective diet score and risk of cardiometabolic abnormalities.

Outcome	N	Events	HR [95% CI]	p-value	Adjusted p-value
**Overweight/Obesity**					
1 SD increase	177712	10763	0.91 [0.89, 0.93]	<0.001	<0.001
Q1 (Ref.)	43986	3358	1.00 (Ref.)		
Q2	44133	2734	0.89 [0.84, 0.93]	<0.001	<0.001
Q3	44822	2464	0.83 [0.79, 0.87]	<0.001	<0.001
Q4	44771	2207	0.77 [0.73, 0.82]	<0.001	<0.001
**Hyperlipidaemia**					
1 SD increase	189404	2681	0.93 [0.89, 0.96]	<0.001	<0.001
Q1 (Ref.)	47604	789	1.00 (Ref.)		
Q2	47072	683	0.92 [0.83, 1.02]	0.133	0.176
Q3	47493	641	0.88 [0.79, 0.98]	0.017	0.033
Q4	47235	568	0.81 [0.72, 0.90]	<0.001	<0.001
**Hypertension**					
1 SD increase	129092	20394	0.97 [0.96, 0.98]	<0.001	<0.001
Q1 (Ref.)	31476	5441	1.00 (Ref.)		
Q2	31893	5213	0.98 [0.95, 1.02]	0.358	0.384
Q3	32776	4878	0.91 [0.88, 0.95]	<0.001	<0.001
Q4	32947	4862	0.93 [0.89, 0.97]	<0.001	<0.001
**Hyperglycaemia/Type 2 Diabetes**					
1 SD increase	180733	8006	0.94 [20.92, 0.96]	<0.001	<0.001
Q1 (Ref.)	44846	2591	1.00 (Ref.)		
Q2	44960	1974	0.87 [0.82, 0.92]	<0.001	<0.001
Q3	45549	1783	0.84 [0.79, 0.89]	<0.001	<0.001
Q4	45378	1658	0.83 [0.78, 0.89]	<0.001	<0.001

Cox proportional hazard models were adjusted by age (timescale), sex, ethnicity, Townsend deprivation index, average house income, education level, dietary supplement use, history of cancer diagnosis, history of hyperglycemia/type 2 diabetes diagnosis (except when hyperglycemia/type 2 diabetes was the outcome), physical activity level, sitting, sleep quality, smoke habits. HR, Hazard Ratio. SD=Standard Deviation.

*Adjusted p-values obtained using Benjamini-Hochberg procedure.

Table S11. Stratified analyses interactions between heart-protective diet score and sex or Townsend deprivation index.

Analysis stratified by sex

	Females				Males					
**Outcome**	**N**	**Events**	**HR [95% CI]**	**p-value**	**N**	**Events**	**HR [95% CI]**	**p-value**	**p-value for the interaction**	**Adjusted p-value for the interaction**
**CVD Incidence**									0.002	0.008
1 SD increase	100869	8103	0.94 [0.92, 0.97]	<0.001	70713	12589	0.98 [0.96, 1.00]	0.041		
Q1 (Ref.)	19878	1724	1.00 (Ref.)		22826	4019	1.00 (Ref.)			
Q2	24250	1952	0.92 [0.86, 0.98]	0.011	18289	3309	0.99 [0.95, 1.04]	0.828		
Q3	27075	2130	0.87 [0.82, 0.93]	<0.001	16125	2849	0.96 [0.91, 1.01]	0.094		
Q4	29666	2297	0.85 [0.79, 0.9]	<0.001	13473	2412	0.96 [0.91, 1.01]	0.128		
**CVD Mortality**									0.962	0.962
1 SD increase	108642	330	0.92 [0.82, 1.03]	0.15	82501	801	0.91 [0.84, 0.97]	0.007		
Q1 (Ref.)	21516	86	1.00 (Ref.)		26566	279	1.00 (Ref.)			
Q2	26139	63	0.6 [0.44, 0.84]	0.002	21389	209	0.91 [0.76, 1.09]	0.321		
Q3	29120	85	0.7 [0.52, 0.95]	0.023	18794	180	0.88 [0.73, 1.06]	0.181		
Q4	31867	96	0.73 [0.54, 0.98]	0.036	15752	133	0.76 [0.62, 0.94]	0.012		
**IHD Incidence**									0.003	0.009
1 SD increase	104956	4016	0.92 [0.89, 0.95]	<0.001	76077	7225	0.97 [0.95, 1.00]	0.026		
Q1 (Ref.)	20691	911	1.00 (Ref.)		24483	2362	1.00 (Ref.)			
Q2	25237	965	0.88 [0.8, 0.97]	0.007	19694	1904	0.99 [0.94, 1.06]	0.846		
Q3	28152	1053	0.85 [0.78, 0.93]	0.001	17346	1628	0.96 [0.9, 1.02]	0.22		
Q4	30876	1087	0.8 [0.73, 0.87]	<0.001	14554	1331	0.93 [0.87, 1]	0.054		
**IHD Mortality**									0.598	0.747
1 SD increase	108865	107	0.85 [0.7, 1.04]	0.122	82896	406	0.88 [0.8, 0.98]	0.016		
Q1 (Ref.)	21570	32	1.00 (Ref.)		26710	135	1.00 (Ref.)			

Q2	26184	18	0.46 [0.26, 0.83]	0.009	21487	111	1.01 [0.79, 1.3]	0.923		
Q3	29174	31	0.7 [0.42, 1.15]	0.159	18881	93	0.95 [0.73, 1.25]	0.725		
Q4	31937	26	0.54 [0.32, 0.93]	0.025	15818	67	0.81 [0.6, 1.09]	0.164		
**MI Incidence**									0.792	0.848
1 SD increase	107956	1016	0.93 [0.87, 0.99]	0.033	80866	2436	0.94 [0.9, 0.98]	0.004		
Q1 (Ref.)	21375	227	1.00 (Ref.)		26002	843	1.00 (Ref.)			
Q2	25955	247	0.92 [0.77, 1.11]	0.381	20940	658	0.99 [0.89, 1.1]	0.831		
Q3	28937	268	0.89 [0.74, 1.06]	0.191	18450	524	0.9 [0.81, 1.01]	0.067		
Q4	31689	274	0.83 [0.69, 1]	0.045	15474	411	0.85 [0.76, 0.96]	0.009		
**MI Mortality**									0.130	0.194
1 SD increase	108910	62	0.73 [0.56, 0.95]	0.020	83072	230	0.93 [0.81, 1.06]	0.264		
Q1 (Ref.)	21580	22	1.00 (Ref.)		26762	83	1.00 (Ref.)			
Q2	26191	11	0.41 [0.2, 0.85]	0.016	21542	56	0.83 [0.59, 1.17]	0.298		
Q3	29191	14	0.44 [0.22, 0.87]	0.018	18920	54	0.9 [0.64, 1.28]	0.562		
Q4	31948	15	0.42 [0.21, 0.83]	0.012	15848	37	0.73 [0.49, 1.08]	0.116		
**Stroke Incidence**									0.792	0.848
1 SD increase	107671	1301	1.01 [0.95, 1.07]	0.783	81500	1802	0.95 [0.91, 1.00]	0.032		
Q1 (Ref.)	21358	244	1.00 (Ref.)		26236	609	1.00 (Ref.)			
Q2	25880	322	1.08 [0.91, 1.27]	0.379	21144	454	0.9 [0.79, 1.01]	0.083		
Q3	28868	337	0.98 [0.83, 1.16]	0.809	18595	379	0.84 [0.74, 0.95]	0.008		
Q4	31565	398	1.04 [0.89, 1.23]	0.603	15525	360	0.94 [0.82, 1.07]	0.338		
**Stroke Mortality**									0.130	0.194
1 SD increase	108814	158	1.09 [0.93, 1.29]	0.300	83109	193	0.9 [0.78, 1.04]	0.157		
Q1 (Ref.)	21572	30	1.00 (Ref.)		26773	72	1.00 (Ref.)			

Q2	26173	29	0.79 [0.48, 1.33]	0.379	21548	50	0.8 [0.56, 1.16]	0.238		
Q3	29160	45	1.06 [0.66, 1.69]	0.820	18935	39	0.69 [0.46, 1.02]	0.064		
Q4	31909	54	1.15 [0.73, 1.81]	0.553	15853	32	0.65 [0.43, 1]	0.048		
**HF Incidence**									0.017	0.036
1 SD increase	107738	1234	0.89 [0.84, 0.95]	<0.001	81347	1955	0.98 [0.93, 1.02]	0.283		
Q1 (Ref.)	21304	298	1.00 (Ref.)		26211	634	1.00 (Ref.)			
Q2	25911	291	0.8 [0.68, 0.95]	0.009	21069	529	1.02 [0.91, 1.15]	0.694		
Q3	28897	308	0.74 [0.63, 0.87]	<0.001	18546	428	0.93 [0.82, 1.05]	0.244		
Q4	31626	337	0.73 [0.62, 0.86]	<0.001	15521	364	0.94 [0.82, 1.07]	0.338		
**HF Mortality**									0.097	0.182
1 SD increase	108932	40	0.73 [0.53, 1.02]	0.065	83201	101	0.89 [0.73, 1.09]	0.257		
Q1 (Ref.)	21586	16	1.00 (Ref.)		26805	40	1.00 (Ref.)			
Q2	26193	9	0.52 [0.23, 1.19]	0.123	21578	20	0.6 [0.35, 1.03]	0.063		
Q3	29197	8	0.44 [0.18, 1.04]	0.060	18948	26	0.87 [0.52, 1.43]	0.573		
Q4	31956	7	0.37 [0.15, 0.92]	0.033	15870	15	0.59 [0.32, 1.08]	0.09		
**AF Incidence**									0.002	0.008
1 SD increase	105538	3434	0.95 [0.92, 0.98]	0.004	77823	5479	1.01 [0.98, 1.04]	0.382		
Q1 (Ref.)	20913	689	1.00 (Ref.)		25207	1638	1.00 (Ref.)			
Q2	25365	837	0.95 [0.86, 1.06]	0.362	20143	1455	1.04 [0.97, 1.12]	0.268		
Q3	28299	906	0.88 [0.8, 0.97]	0.014	17701	1273	1.01 [0.94, 1.09]	0.796		
Q4	30961	1002	0.87 [0.79, 0.96]	0.005	14772	1113	1.04 [0.96, 1.12]	0.331		
**Overweight/Obesity**									<0.001	<0.001
1 SD increase	100666	6105	0.88 [0.86, 0.9]	<0.001	77046	4658	0.95 [0.92, 0.98]	0.001		

Q1 (Ref.)	19409	1646	1.00 (Ref.)		24577	1712	1.00 (Ref.)			
Q2	24131	1534	0.84 [0.79, 0.91]	<0.001	20002	1200	0.93 [0.86, 1]	0.051		
Q3	27161	1503	0.79 [0.74, 0.85]	<0.001	17661	961	0.87 [0.8, 0.94]	0.001		
Q4	29965	1422	0.71 [0.66, 0.77]	<0.001	14806	785	0.88 [0.81, 0.96]	0.004		
**Hyperlipidaemia**									0.311	0.424
1 SD increase	107765	1116	0.91 [0.86, 0.97]	0.004	81639	1565	0.94 [0.89, 0.99]	0.015		
Q1 (Ref.)	21331	254	1.00 (Ref.)		26273	535	1.00 (Ref.)			
Q2	25902	279	0.92 [0.78, 1.09]	0.339	21170	404	0.92 [0.81, 1.05]	0.228		
Q3	28902	277	0.81 [0.68, 0.96]	0.018	18591	364	0.93 [0.82, 1.07]	0.32		
Q4	31630	306	0.81 [0.69, 0.97]	0.018	15605	262	0.8 [0.69, 0.93]	0.003		
**Hypertension**									0.006	0.015
1 SD increase	77785	10394	0.95 [0.93, 0.97]	<0.001	51307	10000	0.99 [0.97, 1.01]	0.242		
Q1 (Ref.)	14965	2171	1.00 (Ref.)		16511	3270	1.00 (Ref.)			
Q2	18596	2601	0.96 [0.91, 1.02]	0.203	13297	2612	0.99 [0.94, 1.04]	0.723		
Q3	21084	2644	0.86 [0.81, 0.91]	<0.001	11692	2234	0.97 [0.92, 1.02]	0.279		
Q4	23140	2978	0.88 [0.83, 0.93]	<0.001	9807	1884	0.97 [0.92, 1.03]	0.370		
**Hyperglycaemia/Type 2 Diabetes**									<0.001	<0.001
1 SD increase	104133	3437	0.88 [0.85, 0.91]	<0.001	76600	4569	0.98 [0.95, 1.01]	0.144		
Q1 (Ref.)	20335	926	1.00 (Ref.)		24511	1665	1.00 (Ref.)			
Q2	25035	829	0.8 [0.73, 0.88]	<0.001	19925	1145	0.91 [0.84, 0.98]	0.013		
Q3	28018	840	0.77 [0.7, 0.84]	<0.001	17531	943	0.88 [0.81, 0.96]	0.002		
Q4	30745	842	0.71 [0.65, 0.79]	<0.001	14633	816	0.94 [0.87, 1.03]	0.182		

Analysis stratified by Townsend Deprivation Index

	< 2.6 (Median value)				≥ 2.6 (Median value)					
Outcome	N	Events	HR [95% CI]	p-value	N	Events	HR [95% CI]	p-value	p-value for the interaction	Adjusted p-value for the interaction
**CVD Incidence**									0.051	0.165
1 SD increase	85654	10466	0.98 [0.96, 1]	0.063	85928	10226	0.95 [0.93, 0.97]	<0.001		
Q1 (Ref.)	20631	2765	1.00 (Ref.)		22073	2978	1.00 (Ref.)			
Q2	21480	2688	0.99 [0.94, 1.04]	0.64	21059	2573	0.96 [0.91, 1.01]	0.104		
Q3	22028	2586	0.96 [0.91, 1.01]	0.114	21172	2393	0.91 [0.86, 0.96]	0.001		
Q4	21515	2427	0.96 [0.9, 1.01]	0.122	21624	2282	0.88 [0.83, 0.93]	<0.001		
**CVD Mortality**									0.053	0.165
1 SD increase	95597	523	0.98 [0.9, 1.08]	0.74	95546	608	0.85 [0.79, 0.93]	<0.001		
Q1 (Ref.)	23244	152	1.00 (Ref.)		24838	213	1.00 (Ref.)			
Q2	24053	115	0.78 [0.61, 1]	0.05	23475	157	0.87 [0.7, 1.07]	0.174		
Q3	24482	132	0.92 [0.72, 1.16]	0.469	23432	133	0.77 [0.62, 0.96]	0.022		
Q4	23818	124	0.93 [0.73, 1.19]	0.575	23801	105	0.64 [0.51, 0.82]	<0.001		
**IHD Incidence**									0.055	0.165
1 SD increase	90513	5607	0.97 [0.95, 1]	0.048	90520	5634	0.94 [0.92, 0.97]	<0.001		
Q1 (Ref.)	21858	1538	1.00 (Ref.)		23316	1735	1.00 (Ref.)			
Q2	22713	1455	0.98 [0.92, 1.06]	0.681	22218	1414	0.94 [0.87, 1.01]	0.08		
Q3	23251	1363	0.95 [0.88, 1.02]	0.155	22247	1318	0.91 [0.85, 0.98]	0.013		
Q4	22691	1251	0.94 [0.87, 1.01]	0.112	22739	1167	0.83 [0.77, 0.9]	<0.001		
**IHD Mortality**									0.217	0.542
1 SD increase	95901	219	0.96 [0.83, 1.1]	0.557	95860	294	0.83 [0.73, 0.93]	0.001		
Q1 (Ref.)	23339	57	1.00 (Ref.)		24941	110	1.00 (Ref.)			
Q2	24115	53	0.99 [0.68, 1.44]	0.946	23556	76	0.84 [0.63, 1.13]	0.258		
Q3	24551	63	1.23 [0.86, 1.78]	0.256	23504	61	0.73 [0.53, 1.01]	0.055		
Q4	23896	46	1 [0.67, 1.49]	0.994	23859	47	0.61 [0.43, 0.87]	0.007		
**MI Incidence**									0.897	0.920
1 SD increase	94343	1777	0.93 [0.88, 0.98]	0.003	94479	1675	0.95 [0.9, 1]	0.038		

Q1 (Ref.)	22855	541	1.00 (Ref.)		24522	529	1.00 (Ref.)			
Q2	23708	460	0.92 [0.81, 1.04]	0.194	23187	445	1.02 [0.9, 1.16]	0.739		
Q3	24188	426	0.9 [0.79, 1.02]	0.107	23199	366	0.9 [0.78, 1.03]	0.122		
Q4	23592	350	0.81 [0.71, 0.93]	0.004	23571	335	0.88 [0.77, 1.02]	0.083		
**MI Mortality**										
1 SD increase	95992	128	0.77 [0.64, 0.93]	0.006	95990	164	0.97 [0.83, 1.14]	0.735		
Q1 (Ref.)	23351	45	1.00 (Ref.)		24991	60	1.00 (Ref.)		0.858	0.920
Q2	24134	34	0.79 [0.51, 1.24]	0.312	23599	33	0.68 [0.44, 1.05]	0.08		
Q3	24584	30	0.7 [0.44, 1.13]	0.144	23527	38	0.85 [0.56, 1.29]	0.456		
Q4	23923	19	0.48 [0.28, 0.83]	0.009	23873	33	0.82 [0.53, 1.28]	0.381		
**Stroke Incidence**										
1 SD increase	94549	1571	1.03 [0.98, 1.09]	0.253	94622	1532	0.92 [0.87, 0.97]	0.001		
Q1 (Ref.)	23007	389	1.00 (Ref.)		24587	464	1.00 (Ref.)		0.011	0.165
Q2	23772	396	1.02 [0.89, 1.17]	0.782	23252	380	0.9 [0.78, 1.03]	0.128		
Q3	24236	378	0.97 [0.84, 1.12]	0.635	23227	338	0.81 [0.71, 0.94]	0.005		
Q4	23534	408	1.09 [0.95, 1.26]	0.233	23556	350	0.85 [0.74, 0.99]	0.031		
**Stroke Mortality**										
1 SD increase	95940	180	1.13 [0.97, 1.31]	0.127	95983	171	0.84 [0.72, 0.99]	0.033		
Q1 (Ref.)	23345	51	1.00 (Ref.)		25000	51	1.00 (Ref.)		0.395	0.740
Q2	24137	31	0.6 [0.38, 0.94]	0.025	23584	48	0.98 [0.66, 1.46]	0.916		
Q3	24570	44	0.83 [0.55, 1.25]	0.378	23525	40	0.8 [0.53, 1.22]	0.305		
Q4	23888	54	1.07 [0.72, 1.59]	0.744	23874	32	0.62 [0.39, 0.98]	0.040		
**HF Incidence**										
1 SD increase	94578	1542	0.94 [0.9, 1]	0.032	94507	1647	0.94 [0.9, 0.99]	0.023		
Q1 (Ref.)	22957	439	1.00 (Ref.)		24558	493	1.00 (Ref.)		0.920	0.920
Q2	23774	394	0.91 [0.8, 1.05]	0.192	23206	426	0.98 [0.86, 1.12]	0.774		
Q3	24245	369	0.86 [0.75, 0.99]	0.034	23198	367	0.87 [0.75, 0.99]	0.039		
Q4	23602	340	0.85 [0.73, 0.98]	0.026	23545	361	0.87 [0.75, 1]	0.046		
**HF Mortality**									0.616	0.920
1 SD increase	96063	57	0.84 [0.64, 1.1]	0.203	96070	84	0.86 [0.69, 1.07]	0.178		

Q1 (Ref.)	23371	25	1.00 (Ref.)		25020	31	1.00 (Ref.)			
Q2	24161	7	0.3 [0.13, 0.69]	0.005	23610	22	0.87 [0.5, 1.5]	0.608		
Q3	24599	15	0.65 [0.34, 1.24]	0.190	23546	19	0.8 [0.45, 1.44]	0.459		
Q4	23932	10	0.48 [0.23, 1.02]	0.057	23894	12	0.55 [0.28, 1.1]	0.090		
**AF Incidence**										
1 SD increase	91468	4652	0.99 [0.96, 1.02]	0.622	91893	4261	0.98 [0.95, 1.02]	0.312		
Q1 (Ref.)	22202	1194	1.00 (Ref.)		23918	1133	1.00 (Ref.)		0.696	0.920
Q2	22980	1188	1 [0.92, 1.08]	0.909	22528	1104	1.04 [0.96, 1.13]	0.341		
Q3	23441	1173	0.99 [0.91, 1.07]	0.727	22559	1006	0.95 [0.87, 1.03]	0.228		
Q4	22845	1097	0.98 [0.9, 1.07]	0.687	22888	1018	0.96 [0.88, 1.05]	0.364		
**Overweight/Obesity**										
1 SD increase	89663	4724	0.92 [0.89, 0.95]	<0.001	19822	1650	0.87 [0.83, 0.92]	<0.001		
Q1 (Ref.)	21563	1374	1.00 (Ref.)		5348	584	1.00 (Ref.)		0.253	0.542
Q2	22522	1221	0.9 [0.84, 0.98]	0.011	4714	407	0.87 [0.76, 0.98]	0.027		
Q3	23077	1106	0.83 [0.77, 0.9]	<0.001	4778	363	0.82 [0.72, 0.94]	0.004		
Q4	22501	1023	0.82 [0.76, 0.89]	<0.001	4982	296	0.67 [0.58, 0.78]	<0.001		
**Hyperlipidaemia**										
1 SD increase	94797	1223	0.94 [0.89, 1]	0.042	21534	392	0.91 [0.83, 1.01]	0.075		
Q1 (Ref.)	23021	351	1.00 (Ref.)		5966	119	1.00 (Ref.)		0.771	0.920
Q2	23843	299	0.86 [0.74, 1.01]	0.062	5121	112	1.16 [0.89, 1.5]	0.277		
Q3	24287	306	0.89 [0.76, 1.04]	0.144	5149	82	0.85 [0.64, 1.13]	0.266		
Q4	23646	267	0.83 [0.7, 0.98]	0.026	5298	79	0.81 [0.6, 1.09]	0.167		
**Hypertension**										
1 SD increase	64890	10153	0.98 [0.96, 1]	0.049	14258	2390	0.95 [0.92, 0.99]	0.021		
Q1 (Ref.)	15438	2611	1.00 (Ref.)		3736	728	1.00 (Ref.)		0.051	0.165
Q2	16238	2627	0.98 [0.93, 1.04]	0.564	3397	596	0.95 [0.85, 1.06]	0.377		
Q3	16818	2437	0.9 [0.85, 0.96]	<0.001	3488	514	0.84 [0.75, 0.94]	0.003		
Q4	16396	2478	0.96 [0.9, 1.01]	0.119	3637	552	0.88 [0.79, 0.99]	0.037		
**Hyperglycaemia/Type 2 Diabetes**										
1 SD increase	91211	3418	0.93 [0.9, 0.96]	<0.001	20129	1273	0.93 [0.88, 0.98]	0.007	0.754	0.920

Q1 (Ref.)	21942	1071	1.00 (Ref.)		5470	448	1.00 (Ref.)			
Q2	22950	852	0.84 [0.77, 0.92]	<0.001	4836	279	0.81 [0.7, 0.95]	0.007		
Q3	23443	780	0.8 [0.73, 0.88]	<0.001	4818	298	0.94 [0.81, 1.1]	0.441		
Q4	22876	715	0.8 [0.73, 0.89]	<0.001	5005	248	0.82 [0.7, 0.96]	0.015		

Cox proportional hazard model were adjusted by age (timescale), sex (except when represented the stratification factor), ethnicity, Townsend deprivation index (except when represented the stratification factor), average house income, education level, dietary supplement use, history of cancer diagnosis, history of hyperglycemia/type 2 diabetes diagnosis (except when hyperglycemia/type 2 diabetes was the outcome), physical activity level, sitting, sleep quality, smoke habits. AF, Atrial Fibrillation; HPDS, heart-protective diet score; HR, Hazard Ratio. SD=Standard Deviation.

*Adjusted p-values were obtained using Benjamini-Hochberg procedure.

Table S12. Sensitivity analyses: association between quartiles of heart-protective diet score and risk of incident cardiovascular diseases and cardiometabolic abnormalities excluding self-reported diagnoses.

Outcome	Events	HR [95% CI]	p-value
**CVD Incidence**			
1 SD increase	22064	0.96 [0.95, 0.98]	<0.001
Q1 (Ref.)	5663	1.00 (Ref.)	
Q2	5191	0.97 [0.94, 1.01]	0.132
Q3	4890	0.93 [0.89, 0.96]	<0.001
Q4	4612	0.91 [0.87, 0.94]	<0.001
**IHD Incidence**			
1 SD increase	12549	0.95 [0.94, 0.97]	<0.001
Q1 (Ref.)	3263	1.00 (Ref.)	
Q2	2859	0.96 [0.91, 1.01]	0.109
Q3	2665	0.92 [0.88, 0.97]	0.003
Q4	2403	0.88 [0.83, 0.93]	<0.001
**MI Incidence**			
1 SD increase	3450	0.94 [0.91, 0.97]	<0.001
Q1 (Ref.)	1013	1.00 (Ref.)	
Q2	873	0.99 [0.9, 1.08]	0.811
Q3	751	0.9 [0.82, 0.99]	0.034
Q4	652	0.85 [0.77, 0.94]	0.002
**Stroke Incidence**			
1 SD increase	3199	0.97 [0.93, 1]	0.068
Q1 (Ref.)	829	1.00 (Ref.)	
Q2	756	0.96 [0.87, 1.05]	0.367
Q3	690	0.87 [0.79, 0.97]	0.010
Q4	728	0.95 [0.85, 1.05]	0.307
**HF Incidence**			
1 SD increase	3509	0.95 [0.91, 0.98]	0.002
Q1 (Ref.)	929	1.00 (Ref.)	
Q2	815	0.95 [0.86, 1.04]	0.26
Q3	728	0.86 [0.78, 0.95]	0.002
Q4	698	0.86 [0.78, 0.95]	0.004
**AF Incidence**			
1 SD increase	9085	0.98 [0.96, 1]	0.116
Q1 (Ref.)	2271	1.00 (Ref.)	
Q2	2230	1.01 [0.96, 1.07]	0.661
Q3	2117	0.96 [0.91, 1.02]	0.211
Q4	2039	0.96 [0.9, 1.02]	0.168
**Hypertension**			
1 SD increase	19149	0.97 [0.95, 0.98]	<0.001
Q1 (Ref.)	4971	1.00 (Ref.)	
Q2	4731	0.98 [0.94, 1.02]	0.404

Q3	4402	0.91 [0.87, 0.94]	<0.001
Q4	4425	0.93 [0.89, 0.97]	0.001
**Hyperglycaemia/Type 2 Diabetes**			
1 SD increase	8213	0.94 [0.91, 0.96]	<0.001
Q1 (Ref.)	2509	1.00 (Ref.)	
Q2	1916	0.87 [0.82, 0.93]	<0.001
Q3	1734	0.84 [0.79, 0.89]	<0.001
Q4	1603	0.83 [0.78, 0.89]	<0.001

Cox proportional hazard model adjusted by age (timescale), sex, ethnicity, Townsend deprivation index, average house income, education level, dietary supplement use, history of cancer diagnosis, history of hyperglycemia/type 2 diabetes diagnosis (except when hyperglycemia/type 2 diabetes was the outcome), physical activity level, sitting, sleep quality, smoke habits. HR, Hazard Ratio. SD=Standard Deviation.

Table S13. Association between categorized food groups and risk of cardiovascular disease incidence and mortality.

Food Group	Consumption (grams/week)	CVD Incidence		CVD Mortality	
		HR [95% CI]	p-value	HR [95% CI]	p-value
Wholegrains	[30, 90)	0.95 [0.91, 0.99]	0.025	0.95 [0.79, 1.15]	0.614
	[90, 150)	0.95 [0.91, 1.00]	0.044	1.00 [0.82, 1.22]	0.991
	[150, 210)	0.94 [0.89, 0.98]	0.010	0.96 [0.78, 1.19]	0.701
	[210, 270)	0.97 [0.92, 1.02]	0.262	0.84 [0.66, 1.07]	0.160
	≥270	0.97 [0.92, 1.02]	0.287	0.91 [0.72, 1.15]	0.426
Fruits	[20, 100)	0.96 [0.91, 1.01]	0.082	0.79 [0.65, 0.96]	0.020
	[100, 180)	0.95 [0.91, 0.99]	0.026	0.80 [0.66, 0.97]	0.025
	[180, 260)	1.01 [0.96, 1.06]	0.820	0.86 [0.69, 1.05]	0.145
	[260, 340)	0.95 [0.89, 1.01]	0.074	0.86 [0.68, 1.1]	0.232
	≥340	1.00 [0.94, 1.05]	0.889	0.78 [0.61, 1.00]	0.048
Non-starchy Vegetables	[20, 100)	0.94 [0.89, 0.98]	0.009	0.81 [0.66, 1.00]	0.047
	[100, 180)	0.95 [0.90, 1.00]	0.034	0.93 [0.76, 1.14]	0.493
	[180, 260)	0.98 [0.93, 1.04]	0.533	0.87 [0.69, 1.08]	0.209
	[260, 400)	0.95 [0.90, 1.00]	0.053	0.89 [0.71, 1.12]	0.323
	≥400	1.01 [0.96, 1.07]	0.596	1.00 [0.8, 1.26]	0.967
Starchy Vegetables	[19, 38)	0.99 [0.88, 1.1]	0.786	1.17 [0.74, 1.84]	0.513
	[38, 56)	1.04 [0.94, 1.15]	0.489	0.88 [0.55, 1.43]	0.613
	[56, 94)	1.04 [0.97, 1.12]	0.292	1.27 [0.93, 1.72]	0.135
	≥94	0.92 [0.74, 1.14]	0.441	0.49 [0.12, 1.96]	0.313
Nuts & Seeds	[15, 30)	0.93 [0.88, 0.99]	0.020	0.81 [0.61, 1.08]	0.153
	[30, 45)	0.99 [0.93, 1.05]	0.629	1.05 [0.82, 1.34]	0.713
	[45, 60)	0.83 [0.68, 1.02]	0.082	0.35 [0.09, 1.42]	0.144
	≥60	0.89 [0.81, 0.97]	0.009	0.90 [0.61, 1.33]	0.583
Legumes & Beans	[20, 60)	1.01 [0.96, 1.06]	0.798	1.18 [0.96, 1.44]	0.110
	[60, 100)	0.97 [0.94, 1.01]	0.159	1.08 [0.93, 1.26]	0.300
	≥100	1.06 [1.00, 1.11]	0.047	1.08 [0.86, 1.37]	0.503
Uncoated Fish & Seafood	[70,210)	1.01 [0.98, 1.05]	0.569	1.06 [0.92, 1.24]	0.424
	≥210	1.03 [0.96, 1.11]	0.415	0.79 [0.55, 1.12]	0.185
Eggs	[60,180)	0.96 [0.92, 1.00]	0.040	0.97 [0.82, 1.14]	0.675

	[180,300)	1.05 [0.99, 1.11]	0.078	1.11 [0.88, 1.38]	0.380
	≥300	1.06 [0.91, 1.24]	0.439	0.54 [0.22, 1.31]	0.173
(Reduced-fat and/or No Added Sugar) Milk & Dairy Products	[125, 250)	0.99 [0.92, 1.07]	0.873	1.36 [1.03, 1.80]	0.032
	[250, 375)	1.03 [0.98, 1.09]	0.228	0.95 [0.74, 1.22]	0.706
	≥375	1.05 [0.95, 1.16]	0.323	1.09 [0.72, 1.66]	0.681
Tea, Coffee & Other Low-calorie Drinks	[125, 375)	0.92 [0.88, 0.96]	<0.001	0.92 [0.76, 1.11]	0.371
	[375, 625)	0.92 [0.88, 0.96]	<0.001	0.80 [0.66, 0.98]	0.027
	[625, 875)	0.89 [0.85, 0.93]	<0.001	0.89 [0.72, 1.09]	0.241
	[875, 1375)	0.94 [0.90, 0.99]	0.011	1.03 [0.85, 1.25]	0.758
	≥1375	0.99 [0.94, 1.04]	0.616	0.96 [0.79, 1.17]	0.681
Homemade soup	[125, 500)	1.04 [0.99, 1.1]	0.089	0.99 [0.72, 1.13]	0.359
	≥500	0.99 [0.86, 1.14]	0.845	0.61 [0.29, 1.30]	0.201
Refined Grains	[30, 90)	1.02 [0.98, 1.06]	0.290	0.86 [0.73, 1.01]	0.059
	[90, 150)	1.02 [0.97, 1.06]	0.514	1.09 [0.90, 1.32]	0.357
	[150, 210)	1.03 [0.97, 1.10]	0.297	0.87 [0.67, 1.13]	0.284
	≥210	1.09 [1.03, 1.15]	0.003	1.09 [0.87, 1.37]	0.438
Potatoes	[19, 94)	1.04 [1.00, 1.07]	0.024	1.12 [0.98, 1.29]	0.097
	[94,131)	1.03 [0.95, 1.12]	0.511	0.91 [0.62, 1.34]	0.639
	≥131	1.03 [0.99, 1.08]	0.163	1.01 [0.82, 1.24]	0.950
Meat, Poultry & Processed Meat	[35, 105)	1.04 [1.00, 1.08]	0.026	1.09 [0.93, 1.27]	0.291
	[105, 140)	1.08 [1.00, 1.18]	0.060	1.07 [0.74, 1.55]	0.728
	[140, 175)	1.06 [1.01, 1.11]	0.020	1.03 [0.85, 1.27]	0.739
	≥175	1.05 [1.00, 1.11]	0.040	1.12 [0.91, 1.37]	0.300
Coated Fish & Seafood	[70,210)	1.05 [0.98, 1.13]	0.202	1.22 [0.92, 1.63]	0.170
	≥210	1.06 [0.90, 1.27]	0.477	1.30 [0.67, 2.51]	0.441
(Full-fat and/or Added Sugar) Milk & Dairy Products	[125, 375)	0.96 [0.93, 1.00]	0.062	0.88 [0.74, 1.05]	0.146
	[375, 500)	0.97 [0.92, 1.02]	0.246	0.97 [0.78, 1.21]	0.777
	[500, 625)	0.99 [0.95, 1.03]	0.641	0.88 [0.74, 1.05]	0.155
	[625, 750)	0.99 [0.92, 1.05]	0.710	1.13 [0.86, 1.47]	0.389
	≥750	0.98 [0.93, 1.03]	0.385	0.98 [0.8, 1.19]	0.810
Processed Soup	[125, 375)	1.04 [0.99, 1.1]	0.133	1.05 [0.84, 1.30]	0.685
	≥375	0.93 [0.72, 1.19]	0.543	2.06 [1.02, 4.13]	0.043
	[20, 50)	0.96 [0.92, 0.99]	0.020	0.81 [0.69, 0.96]	0.014

Sugar, Sweets & Desserts, Cookies & Pastries	[50, 90)	0.94 [0.9, 0.98]	0.005	0.92 [0.76, 1.1]	0.359
	[90, 130)	1.00 [0.95, 1.05]	0.958	1.11 [0.9, 1.36]	0.348
	≥130	0.98 [0.93, 1.03]	0.359	0.99 [0.82, 1.21]	0.951
Savoury Snacks	[15, 30)	1.01 [0.91, 1.12]	0.894	1.27 [0.84, 1.92]	0.261
	[30, 45)	1.00 [0.91, 1.09]	0.929	1.12 [0.75, 1.66]	0.584
	≥45	1.10 [0.91, 1.33]	0.337	0.58 [0.19, 1.8]	0.346
Sugary Drinks	[75, 225)	1.00 [0.95, 1.06]	0.875	0.95 [0.75, 1.2]	0.669
	≥225	1.13 [1.00, 1.27]	0.043	0.99 [0.57, 1.72]	0.976
Artificial sweetener	[1, 5)	1.10 [1.04, 1.17]	0.001	1.02 [0.79, 1.31]	0.884
	[5, 9)	1.17 [1.1, 1.25]	<0.001	1.24 [0.96, 1.59]	0.103
	≥9	1.23 [1.14, 1.32]	<0.001	1.49 [1.13, 1.97]	0.005
Unhealthy Fat	[5, 15)	1.00 [0.96, 1.04]	0.907	0.96 [0.8, 1.15]	0.667
	[15, 25)	1.01 [0.96, 1.07]	0.636	0.94 [0.74, 1.19]	0.618
	≥25	1.03 [0.97, 1.11]	0.328	1.28 [0.98, 1.66]	0.066

Cox proportional hazard models were adjusted by age (timescale), sex, ethnicity, Townsend deprivation index, average house income, education level, dietary supplement use, history of cancer diagnosis, history of hyperglycemia/type 2 diabetes diagnosis, physical activity level, sitting, sleep quality, smoke habits.

CVD: Cardiovascular Disease.

Table S14. Food groups and nutrient intake of LIVEPLUS participants at baseline based on quartile categories of heart-protective diet scores

**Quartiles**	**Q1**	**Q2**	**Q3**	**Q4**
Heart-protective diet score	-14.0	0.3	11.2	36.0
**Food groups intake, servings/d**				
Wholegrains	0.3 (0.1)	1.8 (0.9)	2.7 (1.1)	3.3 (1.2)
Fruits	0.1 (0.1)	0.7 (0.3)	1.4 (0.5)	4.8 (1.6)
Vegetables	2.2 (0.2)	4.6 (1.2)	5.3 (1.5)	6.9 (1.8)
Starchy Vegetables	0.3 (0.2)	0.6 (0.2)	0.7 (0.3)	2.5 (0.9)
Nuts & Seeds	0.4 (0.3)	0.7 (0.3)	1.1 (0.4)	2.3 (0.8)
Legumes & Beans, Other Vegetarian Protein Alternatives	0.0 (0.0)	0.0 (0.0)	0.3 (0.1)	1.0 (0.3)
Seafood with high omega-3 fatty acids	0.1 (0.0)	0.2 (0.1)	0.3 (0.1)	1.0 (0.3)
Seafood with low omega-3 fatty acids	0.0 (0.0)	0.2 (0.1)	0.2 (0.1)	0.9 (0.3)
Eggs	0.1 (0.0)	0.1 (0.0)	0.2 (0.1)	0.5 (0.1)
Milk & Dairy Products	1.5 (0.6)	1.9 (0.7)	2.4 (0.8)	4.7 (1.3)
Refined grains & cereals	5.9 (1.9)	5.0 (1.5)	2.5 (0.9)	1.9 (0.9)
Potatoes	2.5 (0.8)	0.5 (0.2)	0.3 (0.1)	0.0 (0.0)
Red Meats	2.0 (0.7)	1.1 (0.4)	0.5 (0.2)	0.2 (0.1)
Poultry	0.5 (0.2)	0.4 (0.2)	0.2 (0.1)	0.0 (0.0)
Processed Meat	0.4 (0.2)	0.2 (0.1)	0.2 (0.1)	0.0 (0.0)
Solid Fat (tsp/d)	11.1 (2.8)	8.7 (2.1)	6.5 (1.9)	6.0 (2.3)
**Daily nutrient intake**				
Total energy intake, kJ/d	9934.9 (1634.5)	9248.3 (1424.0)	8182.7 (1169.1)	6601.8 (611.9)
Total energy intake, kcal/d	2376.8 (391.0)	2212.5 (340.7)	1957.6 (279.7)	1579.4 (146.4)
Total fat, g/d	120.2 (21.8)	89.3 (13.1)	75.4 (11.1)	67.5 (11.1)
Saturated fatty acids, g/d	34.6 (7.0)	34.3 (6.3)	26.3 (4.4)	23.1 (3.8)
Trans fatty acids, g/d	3.5 (0.8)	1.6 (0.3)	1.2 (0.2)	1.0 (0.2)
Dietary fibre, g/d	19.5 (6.7)	26.8 (7.3)	28.6 (7.6)	36.5 (8.5)
Sodium, mg/d	3267.8 (777.9)	2840.1 (635.7)	2376.5 (477.4)	1433.6 (139.7)

Table S15. Food groups and nutrient intake LIVEPLUS participants at one month based on quartile categories of heart-protective diet scores

Quartiles	Q1	Q2	Q3	Q4
Heart-protective diet score	-7.5	4.8	14.5	35.8
**Food groups intake, servings/d**				
Wholegrains	0.7 (0.3)	1.6 (0.6)	1.9 (0.7)	3.4 (1.0)
Fruits	0.2 (0.1)	0.7 (0.3)	1.5 (0.6)	2.1 (0.8)
Vegetables	1.9 (0.7)	2.5 (0.8)	3.5 (1.0)	6.1 (1.7)
Starchy Vegetables	0.0 (0.0)	0.0 (0.0)	0.6 (0.2)	1.4 (0.5)
Nuts & Seeds	0.0 (0.0)	0.1 (0.0)	0.5 (0.2)	4.9 (1.6)
Legumes & Beans, Other Vegetarian Protein Alternatives	0.0 (0.0)	0.0 (0.0)	0.0 (0.0)	1.0 (0.3)
Seafood with high omega-3 fatty acids	0.0 (0.0)	0.2 (0.1)	0.7 (0.2)	1.1 (0.4)
Seafood with low omega-3 fatty acids	0.0 (0.0)	0.1 (0.0)	0.3 (0.1)	1.0 (0.4)
Eggs	0.0 (0.0)	0.2 (0.1)	0.5 (0.2)	0.7 (0.3)
Milk & Dairy Products	1.1 (0.2)	1.4 (0.2)	1.5 (0.2)	2.3 (0.5)
Refined grains & cereals	2.2 (0.7)	1.2 (0.4)	1.0 (0.4)	0.5 (0.3)
Potatoes	1.4 (0.5)	0.3 (0.1)	0.0 (0.0)	0.0 (0.0)
Red Meats	0.5 (0.2)	0.2 (0.1)	0.0 (0.0)	0.0 (0.0)
Poultry	0.6 (0.2)	0.0 (0.0)	0.0 (0.0)	0.0 (0.0)
Processed Meat	0.2 (0.1)	0.0 (0.0)	0.0 (0.0)	0.0 (0.0)
Solid Fat (tsp/d)	8.1 (2.7)	4.6 (1.4)	1.8 (0.5)	1.6 (0.5)
**Daily nutrient intake**				
Total energy intake, kJ/d	7488.8 (1448.4)	6636.5 (1067.2)	5348.8 (690.4)	4381.6 (400.2)
Total energy intake, kcal/d	1791.6 (346.5)	1587.7 (255.3)	1279.6 (165.2)	1048.2 (95.7)
Total fat, g/d	170.6 (43.4)	77.9 (17.8)	64.1 (11.5)	42.8 (3.9)
Saturated fatty acids, g/d	25.4 (5.3)	17.0 (2.7)	14.9 (2.2)	11.9 (1.2)
Trans fatty acids, g/d	1.5 (0.4)	0.8 (0.2)	0.6 (0.2)	0.4 (0.1)
Dietary fibre, g/d	32.0 (8.3)	25.6 (5.5)	15.5 (2.6)	13.0 (1.6)
Potassium, mg/d	1949.1 (243.8)	2322.8 (326.3)	3126.3 (601.0)	3687.5 (772.8)

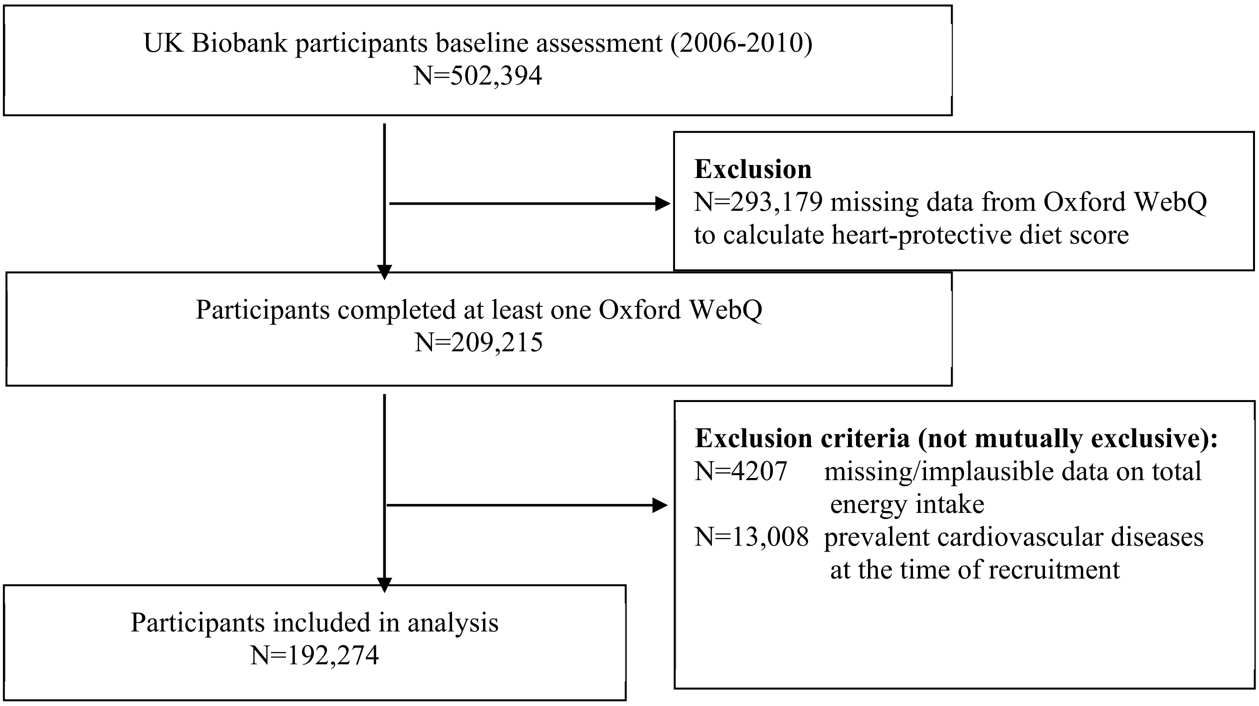

Figure S1. Flow diagram of participants included in the study.

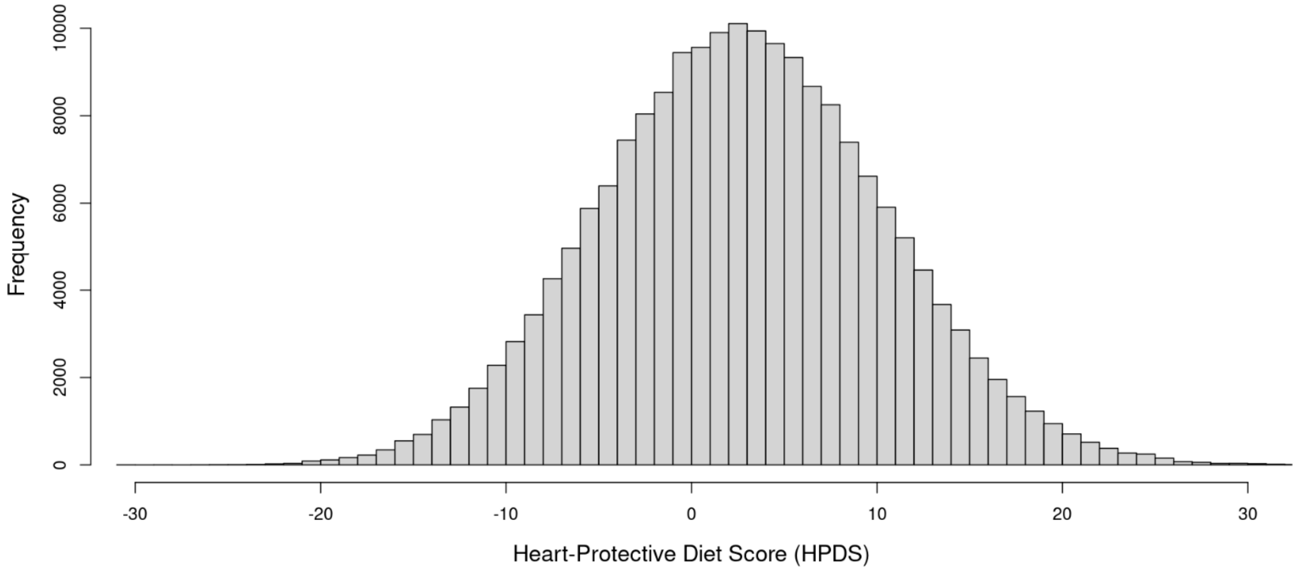

Figure S2. Distribution of the heart-protective diet score

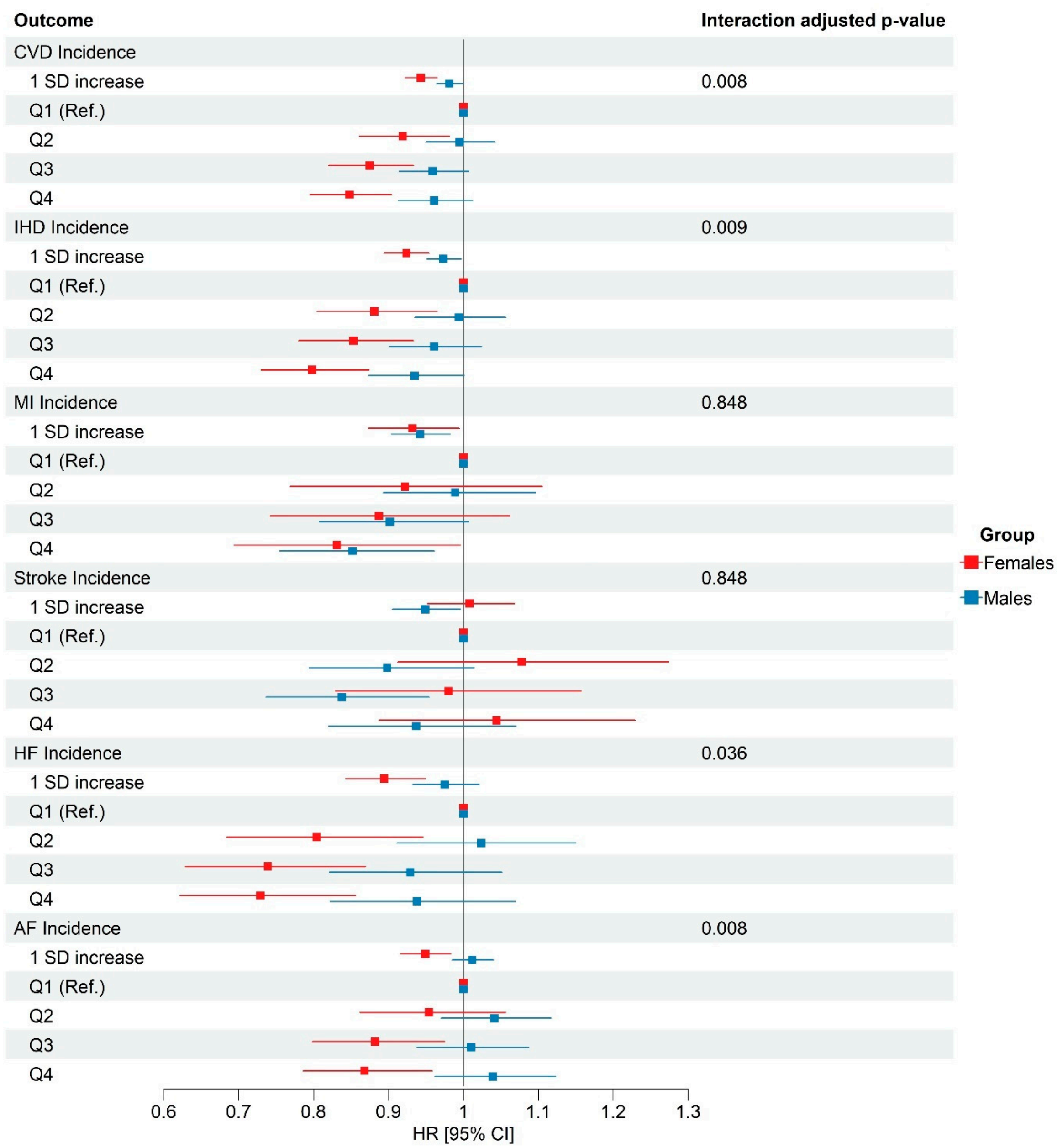
Outcome
Interaction adjusted p-value
CVD Incidence
1 SD increase
0.008
Q1 (Ref.)
Q2
Q3
Q4
IHD Incidence
0.009
1 SD increase
Q1 (Ref.)
Q2
Q3
Q4
MI Incidence
0.848
1 SD increase
Q1 (Ref.)
Q2
Q3
Q4
Stroke Incidence
0.848
1 SD increase
Q1 (Ref.)
Q2
Q3
Q4
HF Incidence
0.036
1 SD increase
Q1 (Ref.)
Q2
Q3
Q4
AF Incidence
0.008
1 SD increase
Q1 (Ref.)
Q2
Q3
Q4
0.6
0.7
0.8
0.9
1
1.1
1.2
1.3
HR [95% CI]
Group
Females
Males

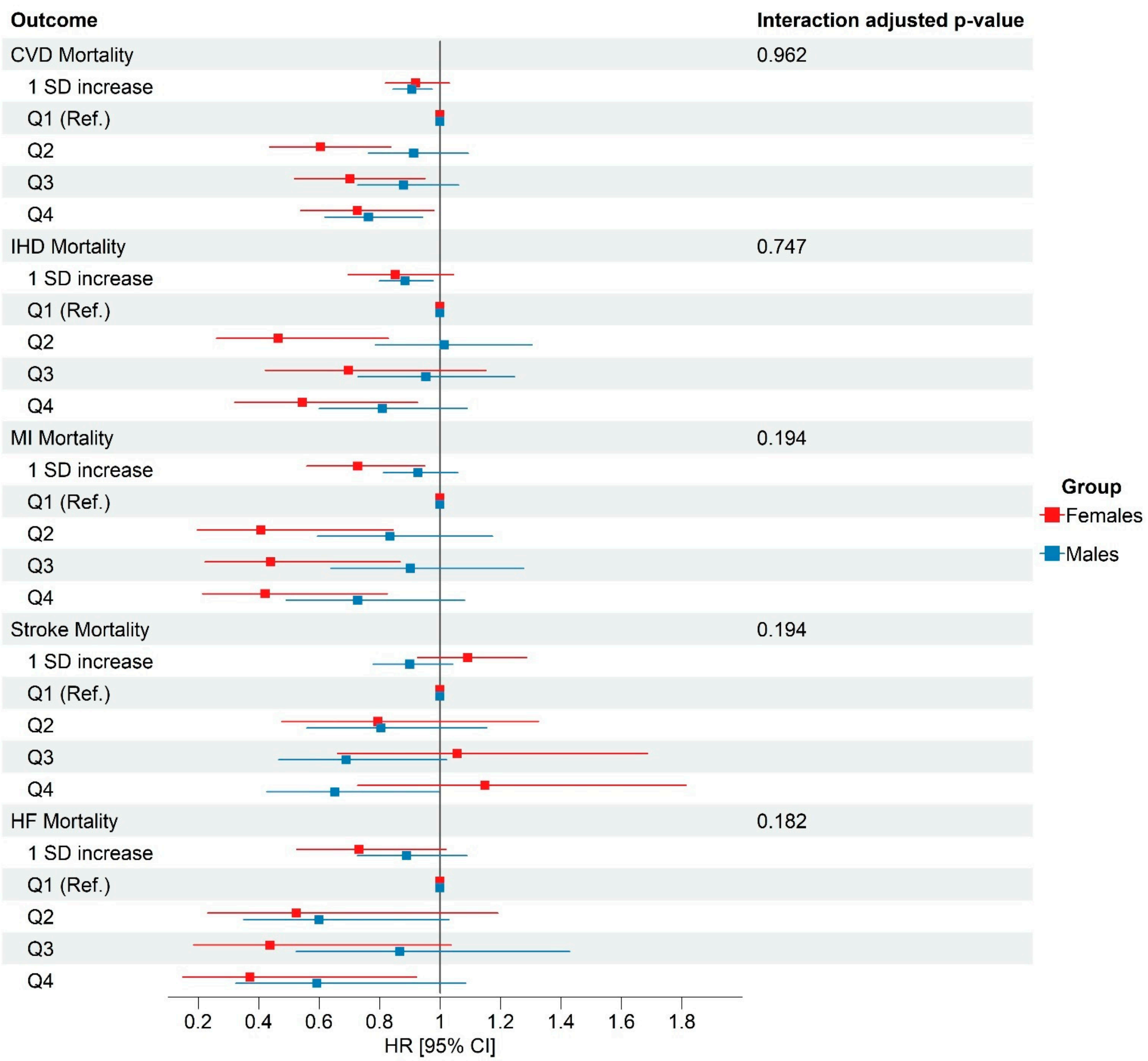
Outcome
Interaction adjusted p-value
CVD Mortality
0.962
1 SD increase
Q1 (Ref.)
Q2
Q3
Q4
IHD Mortality
0.747
1 SD increase
Q1 (Ref.)
Q2
Q3
Q4
MI Mortality
0.194
1 SD increase
Q1 (Ref.)
Q2
Q3
Q4
Stroke Mortality
0.194
1 SD increase
Q1 (Ref.)
Q2
Q3
Q4
HF Mortality
0.182
1 SD increase
Q1 (Ref.)
Q2
Q3
Q4
0.2 0.4 0.6 0.8 1 1.2 1.4 1.6 1.8
HR [95% CI]
Group
Females
Males

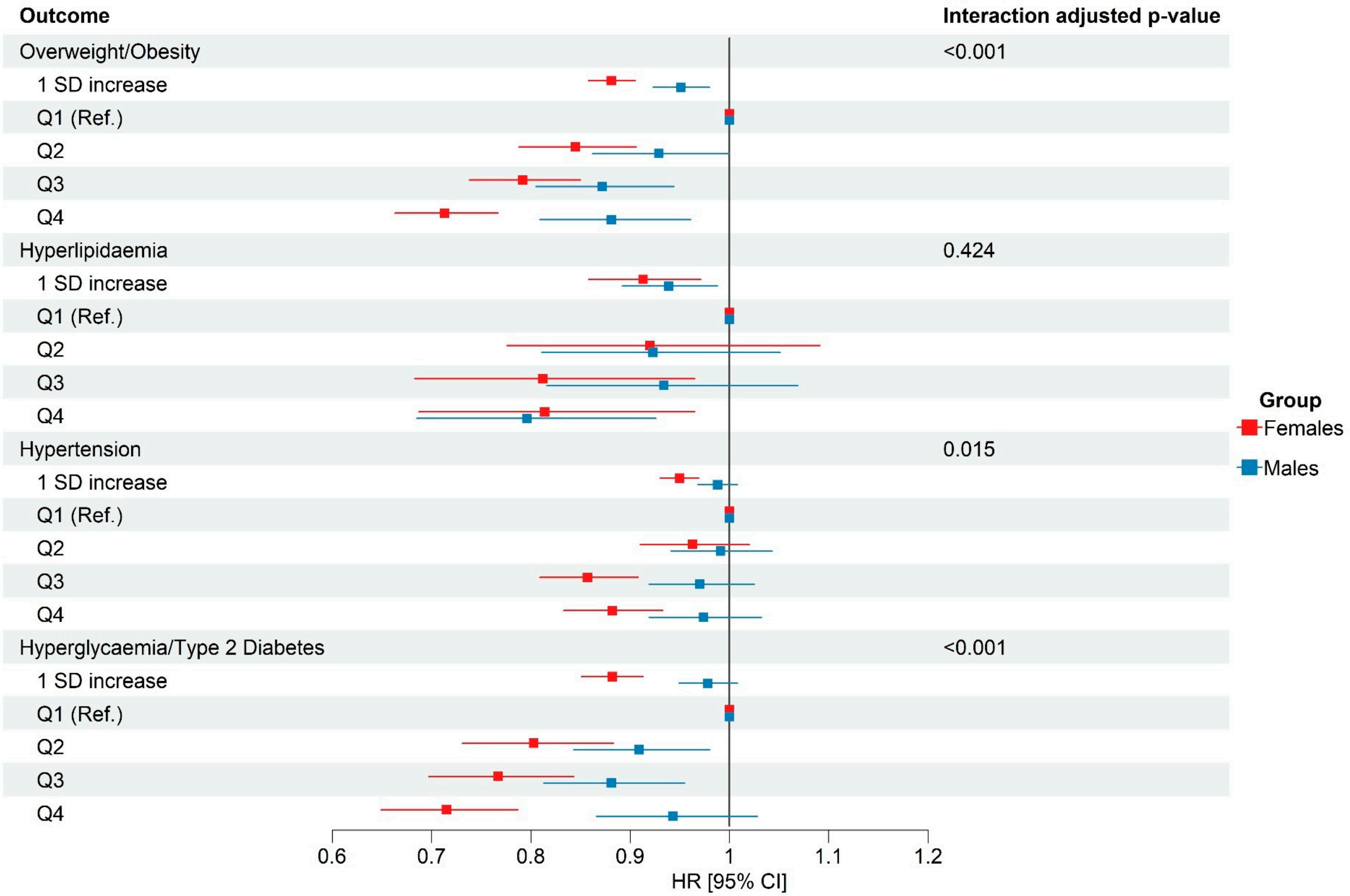

Outcome
Interaction adjusted p-value
Overweight/Obesity
<0.001
1 SD increase
Q1 (Ref.)
Q2
Q3
Q4
Hyperlipidaemia
0.424
1 SD increase
Q1 (Ref.)
Q2
Q3
Q4
Hypertension
0.015
1 SD increase
Q1 (Ref.)
Q2
Q3
Q4
Hyperglycaemia/Type 2 Diabetes
<0.001
1 SD increase
Q1 (Ref.)
Q2
Q3
Q4
0.6
0.7
0.8
0.9
1
1.1
1.2
HR [95% CI]
Group
Females
Males

Figure S3. Stratified analyses for interactions between heart-protective diet score and sex. Data presented as adjusted hazard ratio [95% confidence interval]. Q1 was used as the reference group. Cox proportional hazard models were adjusted by age (timescale), sex (except when represented the stratification factor), ethnicity, Townsend deprivation index (except when represented the stratification factor), average house income, education level, dietary supplement use, history of cancer diagnosis, history of hyperglycemia/type 2 diabetes diagnosis (except when hyperglycemia/type 2 diabetes was the outcome), physical activity level, sitting, sleep quality, smoke habits. HR, Hazard Ratio. Adjusted p-values were obtained using Benjamini-Hochberg procedure.

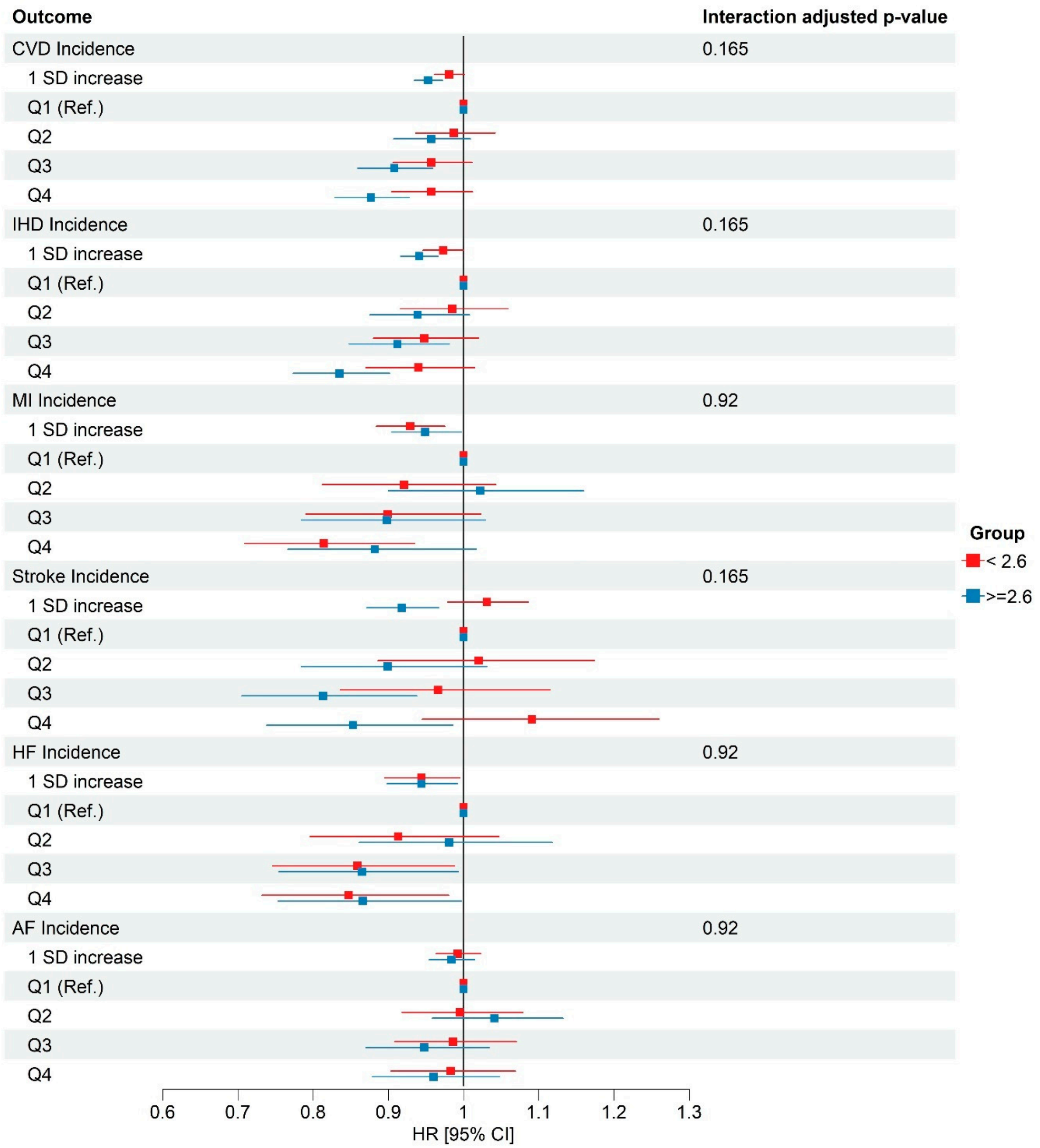
Outcome
Interaction adjusted p-value
CVD Incidence
0.165
1 SD increase
Q1 (Ref.)
Q2
Q3
Q4
IHD Incidence
0.165
1 SD increase
Q1 (Ref.)
Q2
Q3
Q4
MI Incidence
0.92
1 SD increase
Q1 (Ref.)
Q2
Q3
Q4
Stroke Incidence
0.165
1 SD increase
Q1 (Ref.)
Q2
Q3
Q4
HF Incidence
0.92
1 SD increase
Q1 (Ref.)
Q2
Q3
Q4
AF Incidence
0.92
1 SD increase
Q1 (Ref.)
Q2
Q3
Q4
0.6
0.7
0.8
0.9
1
1.1
1.2
1.3
HR [95% CI]
Group
< 2.6
>=2.6

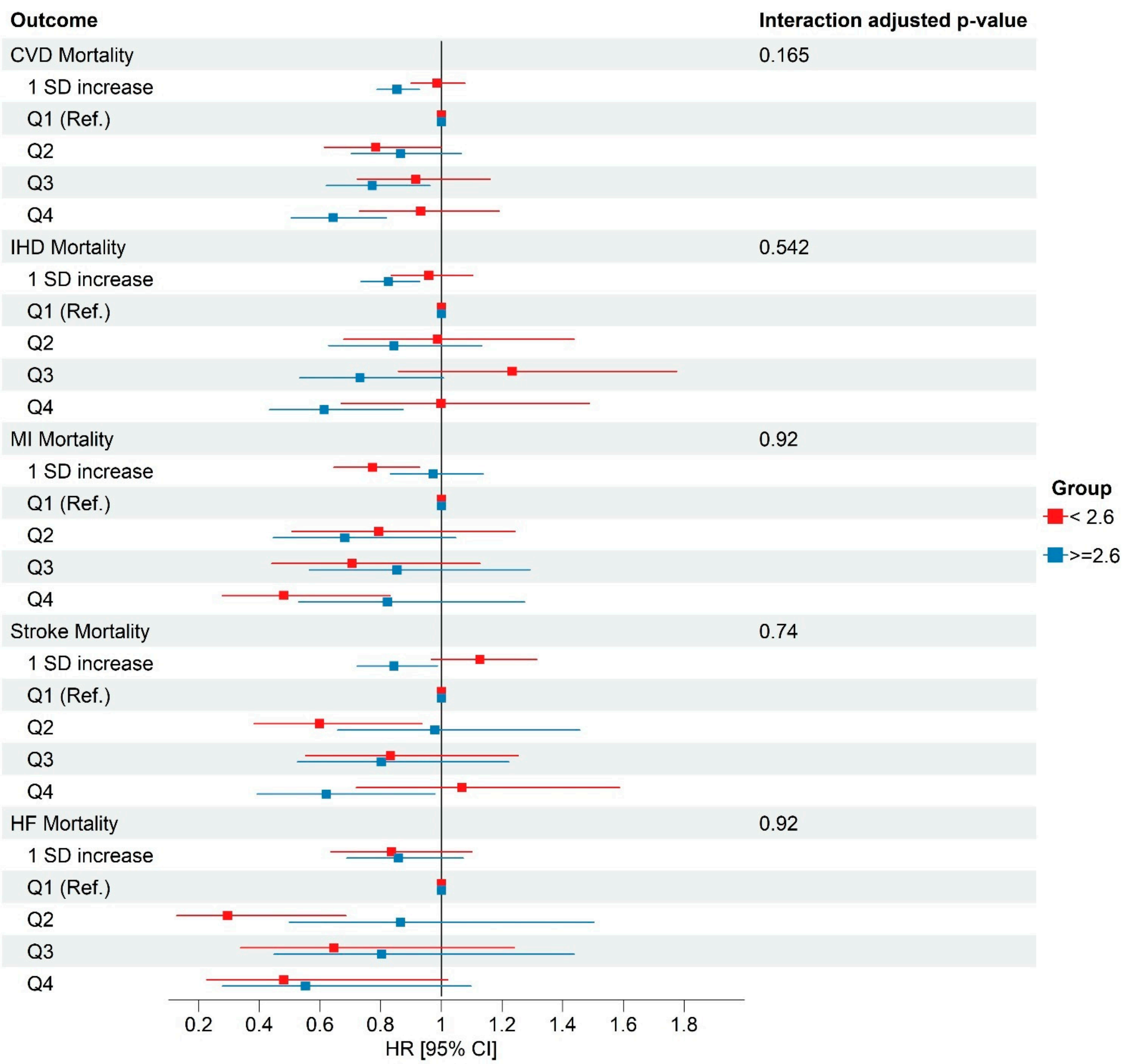
Outcome
Interaction adjusted p-value
CVD Mortality
0.165
1 SD increase
Q1 (Ref.)
Q2
Q3
Q4
IHD Mortality
0.542
1 SD increase
Q1 (Ref.)
Q2
Q3
Q4
MI Mortality
0.92
1 SD increase
Q1 (Ref.)
Q2
Q3
Q4
Stroke Mortality
0.74
1 SD increase
Q1 (Ref.)
Q2
Q3
Q4
HF Mortality
0.92
1 SD increase
Q1 (Ref.)
Q2
Q3
Q4
0.2
0.4
0.6
0.8
1
1.2
1.4
1.6
1.8
HR [95% CI]
Group
< 2.6
>=2.6

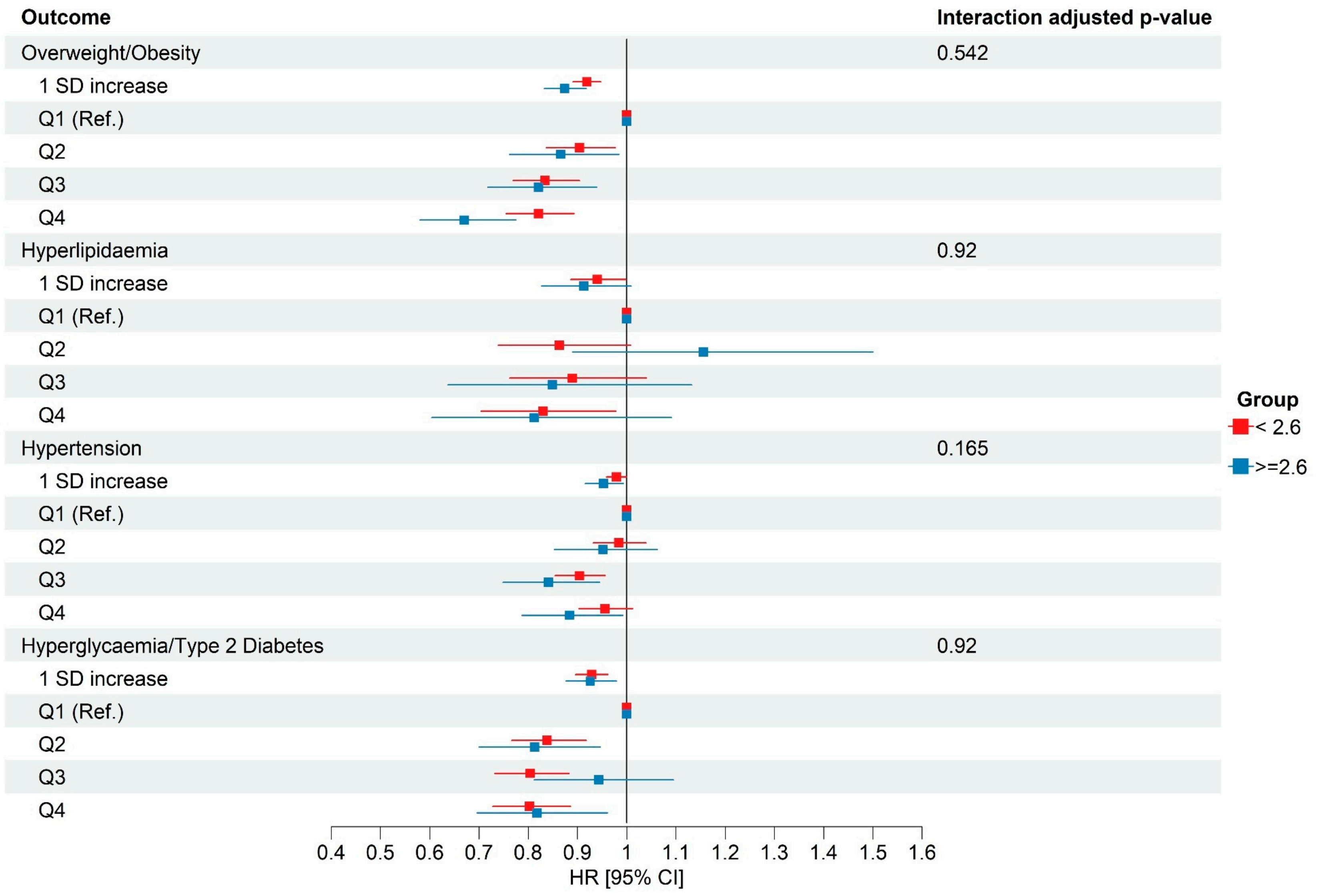
Outcome
Interaction adjusted p-value
Overweight/Obesity
0.542
1 SD increase
Q1 (Ref.)
Q2
Q3
Q4
Hyperlipidaemia
0.92
1 SD increase
Q1 (Ref.)
Q2
Q3
Q4
Hypertension
0.165
1 SD increase
Q1 (Ref.)
Q2
Q3
Q4
Hyperglycaemia/Type 2 Diabetes
0.92
1 SD increase
Q1 (Ref.)
Q2
Q3
Q4
0.4 0.5 0.6 0.7 0.8 0.9 1 1.1 1.2 1.3 1.4 1.5 1.6
HR [95% CI]
Group
< 2.6
>=2.6

Figure S4. Stratified analyses for interactions between heart-protective diet score and Townsend deprivation index. Data presented as adjusted hazard ratio [95% confidence interval]. Q1 was used as the reference group. Cox proportional hazard models were adjusted by age (timescale), sex (except when represented the stratification factor), ethnicity, Townsend deprivation index (except when represented the stratification factor), average house income, education level, dietary supplement use, history of cancer diagnosis, history of hyperglycemia/type 2 diabetes diagnosis (except when hyperglycemia/type 2 diabetes was the outcome), physical activity level, sitting, sleep quality, smoke habits. HR, Hazard Ratio. SD=Standard Deviation. Adjusted p-values were obtained using Benjamini-Hochberg procedure.

Outcome	HR [95% CI] Incl.	HR [95% CI] Excl.
CVD Incidence		
1 SD increase	0.97 [0.95, 0.98]	0.96 [0.95, 0.98]
Q1 (Ref.)	1.00 (Ref.)	1.00 (Ref.)
Q2	0.97 [0.94, 1.01]	0.97 [0.94, 1.01]
Q3	0.93 [0.90, 0.97]	0.93 [0.89, 0.96]
Q4	0.92 [0.88, 0.95]	0.91 [0.87, 0.94]
IHD Incidence		
1 SD increase	0.96 [0.94, 0.98]	0.95 [0.94, 0.97]
Q1 (Ref.)	1.00 (Ref.)	1.00 (Ref.)
Q2	0.96 [0.91, 1.01]	0.96 [0.91, 1.01]
Q3	0.93 [0.88, 0.98]	0.92 [0.88, 0.97]
Q4	0.89 [0.84, 0.94]	0.88 [0.83, 0.93]
MI Incidence		
1 SD increase	0.94 [0.91, 0.97]	0.94 [0.91, 0.97]
Q1 (Ref.)	1.00 (Ref.)	1.00 (Ref.)
Q2	0.97 [0.89, 1.06]	0.99 [0.9, 1.08]
Q3	0.9 [0.82, 0.99]	0.9 [0.82, 0.99]
Q4	0.85 [0.77, 0.94]	0.85 [0.77, 0.94]
Stroke Incidence		
1 SD increase	0.97 [0.94, 1.01]	0.97 [0.93, 1]
Q1 (Ref.)	1.00 (Ref.)	1.00 (Ref.)
Q2	0.95 [0.87, 1.05]	0.96 [0.87, 1.05]
Q3	0.88 [0.8, 0.98]	0.87 [0.79, 0.97]
Q4	0.96 [0.87, 1.07]	0.95 [0.85, 1.05]
HF Incidence		
1 SD increase	0.94 [0.91, 0.98]	0.95 [0.91, 0.98]
Q1 (Ref.)	1.00 (Ref.)	1.00 (Ref.)
Q2	0.95 [0.86, 1.04]	0.95 [0.86, 1.04]
Q3	0.86 [0.78, 0.95]	0.86 [0.78, 0.95]
Q4	0.86 [0.77, 0.95]	0.86 [0.78, 0.95]
AF Incidence		
1 SD increase	0.99 [0.97, 1.01]	0.98 [0.96, 1]
Q1 (Ref.)	1.00 (Ref.)	1.00 (Ref.)
Q2	1.02 [0.96, 1.08]	1.01 [0.96, 1.07]
Q3	0.97 [0.91, 1.03]	0.96 [0.91, 1.02]
Q4	0.97 [0.91, 1.03]	0.96 [0.9, 1.02]
Hypertension		
1 SD increase	0.97 [0.96, 0.98]	0.97 [0.95, 0.98]
Q1 (Ref.)	1.00 (Ref.)	1.00 (Ref.)
Q2	0.98 [0.95, 1.02]	0.98 [0.94, 1.02]
Q3	0.91 [0.88, 0.95]	0.91 [0.87, 0.94]
Q4	0.93 [0.89, 0.97]	0.93 [0.89, 0.97]
Hyperglycaemia/Type 2 Diabetes		
1 SD increase	0.94 [20.92, 0.96]	0.94 [0.91, 0.96]
Q1 (Ref.)	1.00 (Ref.)	1.00 (Ref.)
Q2	0.87 [0.82, 0.92]	0.87 [0.82, 0.93]
Q3	0.84 [0.79, 0.89]	0.84 [0.79, 0.89]
Q4	0.83 [0.78, 0.89]	0.83 [0.78, 0.89]

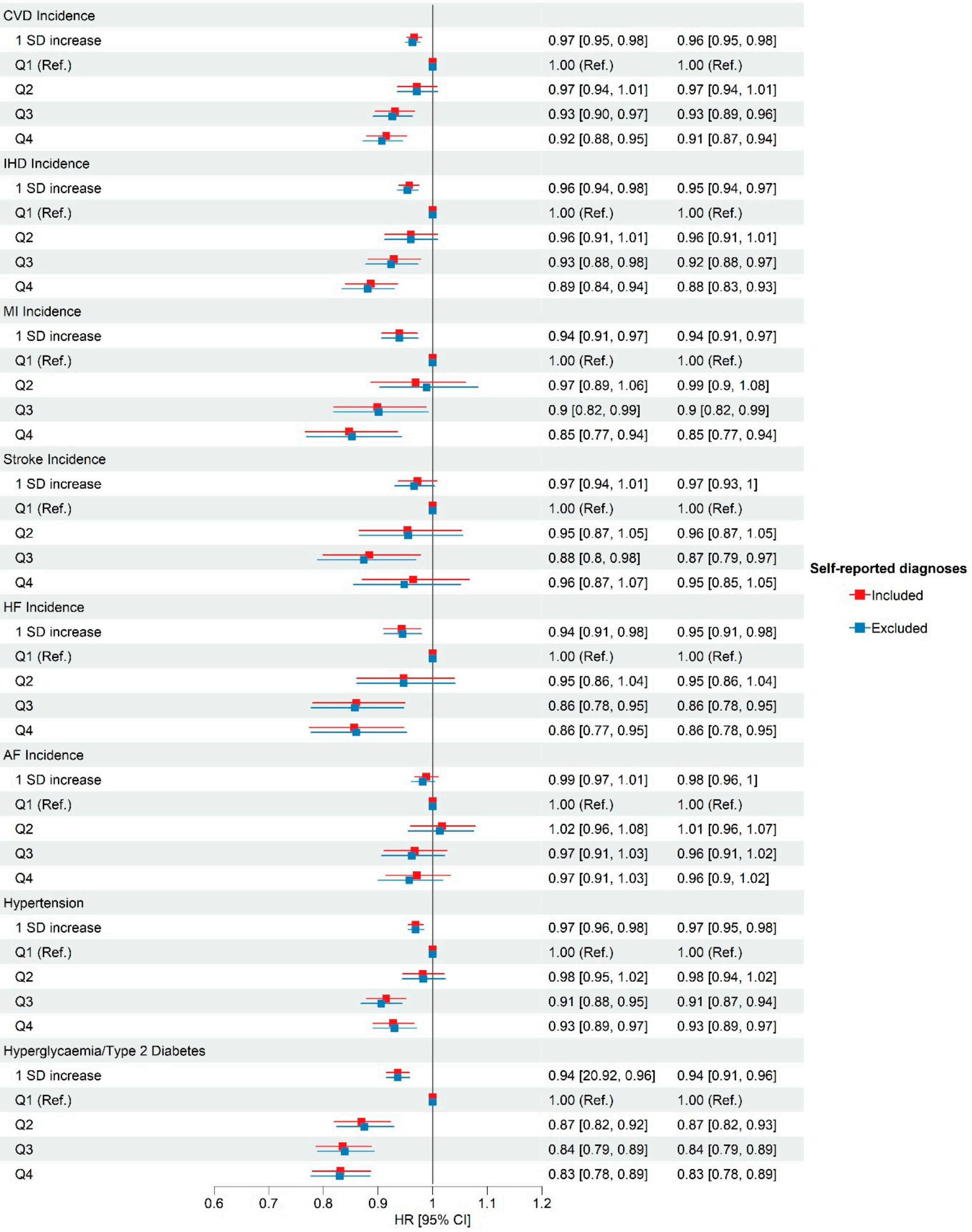

Figure S5. Sensitivity analysis: association between quartiles of heart-protective diet score and risk of incident cardiovascular diseases and cardiometabolic abnormalities including and excluding self-reported diagnoses. Data presented as adjusted hazard ratio [95% confidence interval]. Q1 was used as the reference group. Cox proportional hazard models were adjusted by age (timescale), sex, ethnicity, Townsend deprivation index, average house income, education level, dietary supplement use, history of cancer diagnosis, history of hyperglycemia/type 2 diabetes diagnosis, physical activity level, sitting, sleep quality, smoke habits. CVD, Cardiovascular Disease; IHD, Ischemic Heart Disease; MI, Myocardial Infarction; HF, Heart Failure; AF, Atrial Fibrillation; HR, Hazard Ratio. SD=Standard Deviation. Adjusted p-values were obtained using Benjamini-Hochberg procedure.

References

1. Bycroft C, Freeman C, Petkova D, et al. The UK Biobank resource with deep phenotyping and genomic data. *Nature*. 2018;**562**(7726):203-9.

2. Liu B, Young H, Crowe FL, et al. Development and evaluation of the Oxford WebQ, a low-cost, web-based method for assessment of previous 24 h dietary intakes in large-scale prospective studies. *Public Health Nutr*. 2011;**14**(11):1998-2005.

3. Bradbury KE, Young HJ, Guo W, Key TJ. Dietary assessment in UK Biobank: an evaluation of the performance of the touchscreen dietary questionnaire. *J Nutr Sci*. 2018;**7**:e6.

4. Adams J, Ryan V, White M. How accurate are Townsend Deprivation Scores as predictors of self-reported health? A comparison with individual level data. *J Public Health (Oxf)*. 2005;**27**(1):101-6.

5. Guidelines for data processing and analysis of the International Physical Activity Questionnaire (IPAQ) 2005 [Available from: http://www.IPAQ.ki.se.

6. Craig CL, Marshall AL, Sjöström M, et al. International physical activity questionnaire: 12-country reliability and validity. *Med Sci Sports Exerc*. 2003;**35**(8):1381-95.

7. Fan M, Sun D, Zhou T, et al. Sleep patterns, genetic susceptibility, and incident cardiovascular disease: a prospective study of 385 292 UK biobank participants. *Eur Heart J*. 2020;**41**(11):1182-9.

8. Chen F, Ding C, Du M, et al. Plant- and animal-based diet quality and mortality among US adults: a cohort study. *British Journal of Nutrition*. 2021;**125**(12):1405-15.

9. Caivano S, Colugnati FAB, Domene SMÁ. Diet Quality Index associated with Digital Food Guide: update and validation. *Cadernos de Saúde Pública*. 2019;**35**.

10. Bromage S, Batis C, Bhupathiraju SN, et al. Development and Validation of a Novel Food-Based Global Diet Quality Score (GDQS). *The Journal of Nutrition*. 2021;**151**:75S-92S.

11. Satija A, Bhupathiraju SN, Spiegelman D, et al. Healthful and Unhealthful Plant-Based Diets and the Risk of Coronary Heart Disease in U.S. Adults. *J Am Coll Cardiol*. 2017;**70**(4):411-22.
